# Disruption of mitochondria–sarcoplasmic reticulum microdomain connectomics contributes to sinus node dysfunction in heart failure

**DOI:** 10.1073/pnas.2206708119

**Published:** 2022-08-31

**Authors:** Lu Ren, Raghavender R. Gopireddy, Guy Perkins, Hao Zhang, Valeriy Timofeyev, Yankun Lyu, Daphne A. Diloretto, Pauline Trinh, Padmini Sirish, James L. Overton, Wilson Xu, Nathan Grainger, Yang K. Xiang, Elena N. Dedkova, Xiao-Dong Zhang, Ebenezer N. Yamoah, Manuel F. Navedo, Phung N. Thai, Nipavan Chiamvimonvat

**Affiliations:** ^a^Division of Cardiovascular Medicine, Department of Internal Medicine, University of California, Davis, CA 95616;; ^b^Stanford Cardiovascular Institute, Stanford University School of Medicine, Stanford, CA 94305;; ^c^Department of Pharmacology, University of California, Davis, CA 95616;; ^d^National Center for Microscopy and Imaging Research, University of California San Diego, La Jolla, CA 92093;; ^e^Department of Physiology and Membrane Biology, University of California, Davis, CA 95616;; ^f^Department of Molecular Biosciences, School of Veterinary Medicine, University of California, Davis, CA 95616;; ^g^Department of Physiology and Cell Biology, University of Nevada, Reno, NV 89557;; ^h^Department of Veterans Affairs, Northern California Health Care System, Mather, CA 95655

**Keywords:** sinoatrial node, mitochondria, heart failure, bradycardia, sinoatrial node dysfunction

## Abstract

The sinoatrial node (SAN), the leading pacemaker region, generates electrical impulses that propagate throughout the heart. SAN dysfunction with bradyarrhythmia is well documented in heart failure (HF). However, the underlying mechanisms are not completely understood. Mitochondria are critical to cellular processes that determine the life or death of the cell. The release of Ca^2+^ from the ryanodine receptors 2 (RyR2) on the sarcoplasmic reticulum (SR) at mitochondria–SR microdomains serves as the critical communication to match energy production to meet metabolic demands. Therefore, we tested the hypothesis that alterations in the mitochondria–SR connectomics contribute to SAN dysfunction in HF. We took advantage of a mouse model of chronic pressure overload–induced HF by transverse aortic constriction (TAC) and a SAN-specific CRISPR-Cas9–mediated knockdown of *mitofusin-2* (*Mfn2*), the mitochondria–SR tethering GTPase protein. TAC mice exhibited impaired cardiac function with HF, cardiac fibrosis, and profound SAN dysfunction. Ultrastructural imaging using electron microscope (EM) tomography revealed abnormal mitochondrial structure with increased mitochondria–SR distance. The expression of Mfn2 was significantly down-regulated and showed reduced colocalization with RyR2 in HF SAN cells. Indeed, SAN-specific *Mfn2* knockdown led to alterations in the mitochondria–SR microdomains and SAN dysfunction. Finally, disruptions in the mitochondria–SR microdomains resulted in abnormal mitochondrial Ca^2+^ handling, alterations in localized protein kinase A (PKA) activity, and impaired mitochondrial function in HF SAN cells. The current study provides insights into the role of mitochondria–SR microdomains in SAN automaticity and possible therapeutic targets for SAN dysfunction in HF patients.

## Significance

The human heart beats 60 to 80 times a minute, which can amount to more than 3 billion heartbeats in one’s lifetime. Each heartbeat is initiated by the sinoatrial node (SAN). SAN dysfunction is a common feature of heart failure (HF). However, the underlying mechanisms are not entirely understood. Here, we demonstrate that disruptions in mitochondria–sarcoplasmic reticulum (SR) connectomics contribute, at least in part, to SAN dysfunction in HF. Electron microscope tomography reveals abnormal mitochondrial structure with increased mitochondria–SR distance. In HF SAN cells, the mitochondria–SR tethering GTPase protein, mitofusin-2 (Mfn2), is significantly down-regulated. Furthermore, SAN-specific *Mfn2* knockdown results in SAN dysfunction. The findings highlight the roles of mitochondria–SR connectomics in SAN dysfunction commonly seen with HF.

Heart failure (HF) is a progressive condition that occurs when the heart no longer generates sufficient cardiac output to meet the metabolic demands of the body (1). Despite the current armamentarium in HF therapies, the 5-y mortality in HF patients remains greater than 50% (2). One of the known complications in HF is bradyarrhythmia from sinoatrial node (SAN) dysfunction, which significantly increases the morbidity and mortality of HF patients (3). Patients diagnosed with HF and SAN dysfunction have an increased risk of sudden cardiac death (2, 3). Hence, it is imperative to understand the mechanistic underpinning of SAN dysfunction in HF to improve clinical outcomes.

The SAN is a highly complex structure consisting of specialized cells that spontaneously fire action potentials (APs), propagating throughout the heart. Its automaticity is orchestrated by ion channels and transporters that contribute to the membrane and Ca^2+^ clocks, collectively known as the “coupled clock” (4, 5). These two cyclical processes are significantly impaired in HF, leading to SAN dysfunction (6), with documented remodeling of ion channels, gap junction channels, Ca^2+^-, Na^+^-, and H^+^-handling proteins, and receptors (7). There is a documented reduction in the hyperpolarization-activated “pacemaker” current (*I*_f_) from a decrease in hyperpolarization-activated and cyclic nucleotide–gated (HCN)2 and HCN4 channel expression (8), and a reduction in the slow component of the delayed rectifier K^+^ current (*I*_Ks_) (9).

Given the critical roles of the mitochondria in energy production and determination of cell survival, the remodeling of the coupled clock may be secondary to or potentiated by alterations in mitochondria in HF. Beat-to-beat alterations in electrochemical gradients within the SAN need to be reestablished by the energy-dependent exchangers, primarily fueled by aerobic respiration from the mitochondria.

Similar to ventricular myocytes, the SAN is endowed with a dense mitochondrial network with a high basal respiratory rate (10). To accomplish their role in energy production, they require constant feedback on the cell’s energetic state (11). Organelle connectomics, or close communication between mitochondria and the sarcoplasmic reticulum (SR), occurs at microdomains, physically established, in part, by mitofusin-2 (Mfn2). Mfn2, a dynamin-like GTPase embedded in the outer mitochondrial membrane, is a key protein involved in tethering the mitochondria and SR, ensuring sufficient energy production for cellular bioenergetics (12). Indeed, a critical interorganelle communication occurs within the mitochondria–SR contact sites, mediated in part by microdomains of reactive oxygen species (ROS), Ca^2+^, and cyclic adenosine monophosphate (cAMP). The SAN’s automaticity is highly dependent on the cyclic changes in Ca^2+^ within the cell (13). Specifically, the release of Ca^2+^ from the ryanodine receptors 2 (RyR2) on the SR at mitochondria–SR microdomains—regions of high, localized Ca^2+^—serves as a critical communication to match energy production and demand (11, 14). Additionally, these microdomains serve as crucial communication hubs for cAMP and ROS signaling (15–17).

Therefore, proper communications with the mitochondria is essential for cellular survival, ensuring adequate energy production to meet the metabolic demands of the SAN. Impaired mitochondrial connectomics, either through injury to the mitochondria or disruption of their microdomains, can significantly affect SAN function. However, to date, little is known regarding the mitochondria–SR cross-talk in SAN cells (SANCs) and the critical roles of this communication in regulating SAN automaticity and dysfunction commonly seen in HF.

The objective of this study is to investigate the structural remodeling that occurs in the SAN mitochondria–SR contact sites and how this influences functional outcome in HF. We used a well-established pressure overload murine model where HF was induced by transverse aortic constriction (TAC) (18). Concurrent with the development of HF, the animals show evidence of SAN dysfunction with sinus bradycardia. High-resolution imaging, transmission electron microscopy (TEM), and electron microscope (EM) tomography demonstrate structurally altered mitochondria with impaired Ca^2+^ and cAMP signaling at the mitochondria–SR microdomains. The expression of Mfn2 is significantly down-regulated and shows reduced colocalization with RyR2 in HF SANCs. Importantly, SAN-specific CRISPR-Cas9–mediated *Mfn2* knockdown (KD) recapitulates SAN dysfunction in HF. The data support the critical roles of mitochondria–SR connectomics in regulating SAN automaticity. Structural and functional remodeling of the mitochondria and their microdomains contribute to the pathogenesis of SAN dysfunction in HF.

## Results

### Preclinical Model of TAC-Induced HF.

**Fig. 1.**
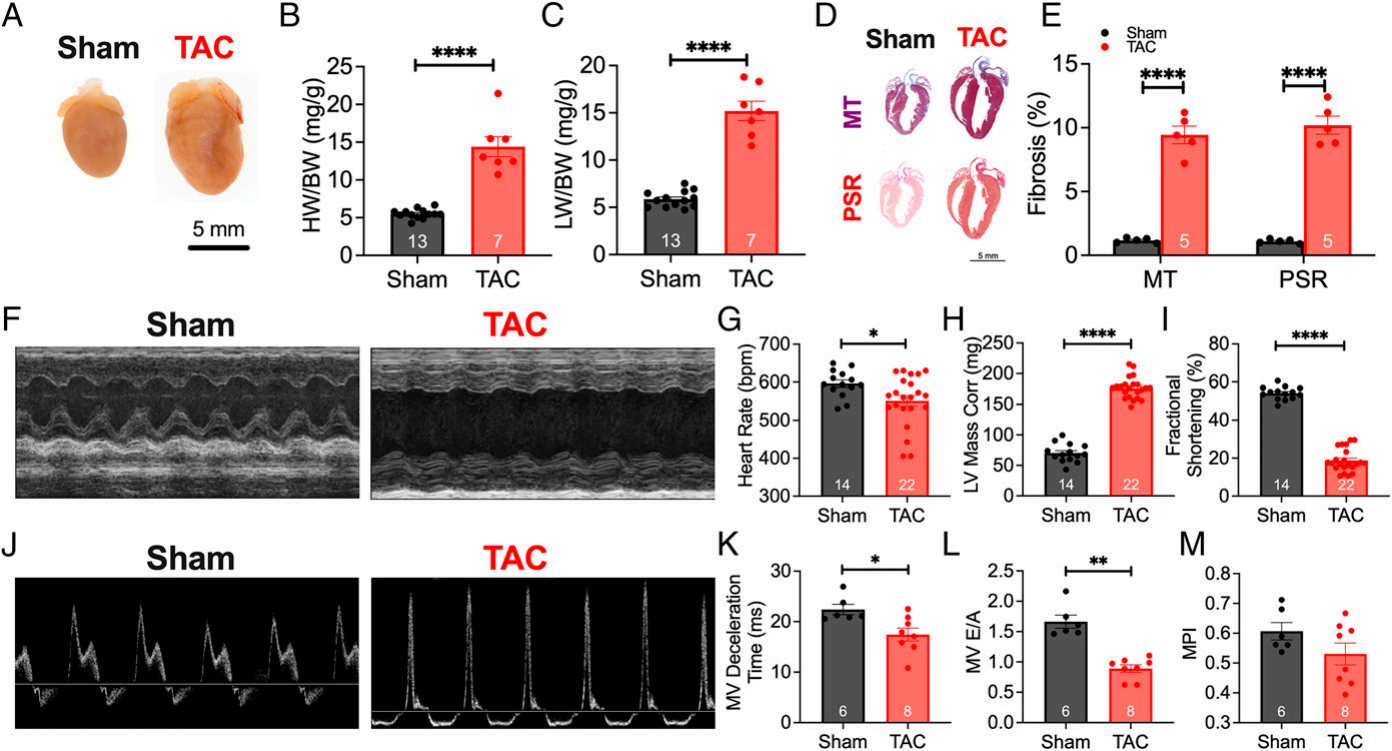
Transverse aortic constriction–induced heart failure. (*A*) Representative images of hearts taken 8 wk after the sham or TAC operations. (*B* and *C*) Summary data of heart weight–to–body weight (HW/BW) (*B*) and lung weight–to–body weight (LW/BW) (*C*) ratios are shown. (*D*) Cardiac sections were stained with Masson’s trichrome and Picrosirius red to assess collagen content, as depicted in the representative images. (*E*) Summary data of collagen deposition for both MT and PSR are shown. Conscious ECG was used to determine cardiac structure and function. (*F*) Representative M-mode images at the parasternal short axis are depicted. (*G*–*I*) Summary data for heart rate (*G*), LV mass–corrected (*H*), and fractional shortening (*I*) are shown. (*J*) Representative images of blood flow through the MV, assessed using pulsed-wave Doppler ECG to quantify diastolic function. (*K*–*M*) Quantification of MV deceleration time (*K*), MV E/A ratio (*L*), and myocardial performance index (MPI) (*M*) are depicted. Data are expressed as mean ± SEM. Gray and red bars are data from sham compared with TAC mice, respectively. *P < 0.05, **P < 0.01, and ****P < 0.0001. The numbers shown within the bar graphs represent the numbers of animals.

SAN dysfunction is well-described in patients with HF (3). We used a well-established pressure overload model to generate the HF model, where the transverse aorta was constricted to a diameter of ∼0.4 mm (Fig. 1). Validation of constriction was performed by taking the ratio of the common carotid arteries’ blood flow velocities (*SI Appendix*, Fig. S1) as previously described (18). Mice were randomly assigned to undergo either sham or TAC surgeries and followed for 8 wk. Representative whole-heart images in Fig. 1*A* show evidence of cardiac dilatation with a significant increase in heart weight/body weight ratios (Fig. 1 *A*, *B*, and *H*; *P* < 0.0001) and pulmonary congestion in the TAC mice (Fig. 1*C*; *P* < 0.0001). Cardiac sections stained with Masson’s trichrome (MT) and Picrosirius red (PSR) (Fig. 1*D*) to detect collagen deposition showed a significant increase in fibrosis in the TAC hearts (Fig. 1*E*; *P* < 0.0001). TAC mice showed evidence of cardiac hypertrophy with a significant increase in left ventricular (LV) mass (Fig. 1*H*; *P* < 0.0001) and depressed systolic function, as seen by the representative M-mode images taken at the parasternal short axis (Fig. 1*F*), and quantitatively by the fractional shortening (Fig. 1*I*; *P* < 0.0001). At this stage of HF, TAC mice exhibited significant sinus bradycardia compared with sham animals (Fig. 1*G*; *P* < 0.05). In addition, diastolic function was assessed in these mice. Representative tracings of blood flow velocity through the mitral valve (MV) are shown in Fig. 1*J*. TAC mice exhibited significantly lower MV deceleration time (Fig. 1*K*; *P* < 0.05) and reduced E wave to A wave (E/A) ratio (Fig. 1*L*; *P* < 0.05), suggesting impaired diastolic function. Myocardial performance index (MPI) was not different between the two groups (Fig. 1*M*). These results demonstrate that after 8 wk of TAC (*SI Appendix*, Table S1), mice developed evidence of structural remodeling with systolic and diastolic dysfunction.

### HF Mice Exhibited Sinus Bradycardia.

**Fig. 2.**
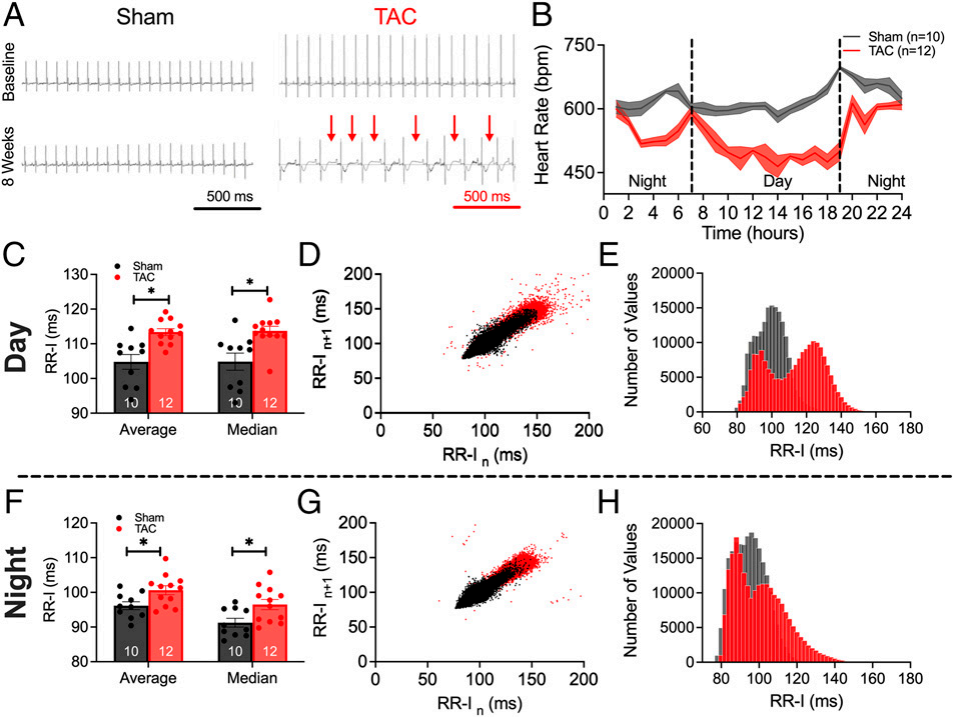
HF mice exhibited sinus bradycardia. (*A*) Representative ECG tracings during the 24-h recording periods at baseline and 8 wk for sham-operated and TAC mice. Red arrows indicate prolonged RR intervals. (*B*) Summary data of the time course of heart rates from 24-h ECG tracings showing the circadian rhythms. bpm, beats per minute. (*C*–*E*) Summary data of average and median RR-I (*C*), scatterplots of HRV (*D*), and histograms of the distribution of RR-I in sham and TAC mice (*E*), measured for daytime hours. (*F*–*H*) Similarly for nighttime in summary data of average and median RR-I (*F*), HRV (*G*), and histograms (*H*). Data are expressed as mean ± SEM. *P < 0.05. Numbers within the bar graphs represent numbers of animals.

Ambulatory echocardiography (ECG) recordings were obtained before surgery and 8 wk after either sham or TAC operations (Fig. 2*A*). TAC mice demonstrated evidence of SAN dysfunction. Summary data for the 24-h recordings reveal a significant reduction in heart rate in HF mice at almost all time points during the circadian rhythm (Fig. 2*B*; *P* < 0.05). The conscious echocardiographic findings corroborated this decrease in heart rate (Fig. 1*G*). Analyses of RR interval (RR-I) during daytime (7 AM to 7 PM; Fig. 2 *C*–*E*) and nighttime (7 PM to 7 AM; Fig. 2 *F*–*H*) revealed a significant elevation in overall RR-I, higher heart rate variability (HRV), and a bimodal distribution of RR-I in HF mice. Together, the data demonstrate abnormal sinus rhythm in HF, with sinus bradycardia at basal heart rate.

### HF SANCs Exhibited Reduced AP Frequency, Impaired Ca^2+^ Transients, and Local Ca^2+^ Release.

**Fig. 3.**
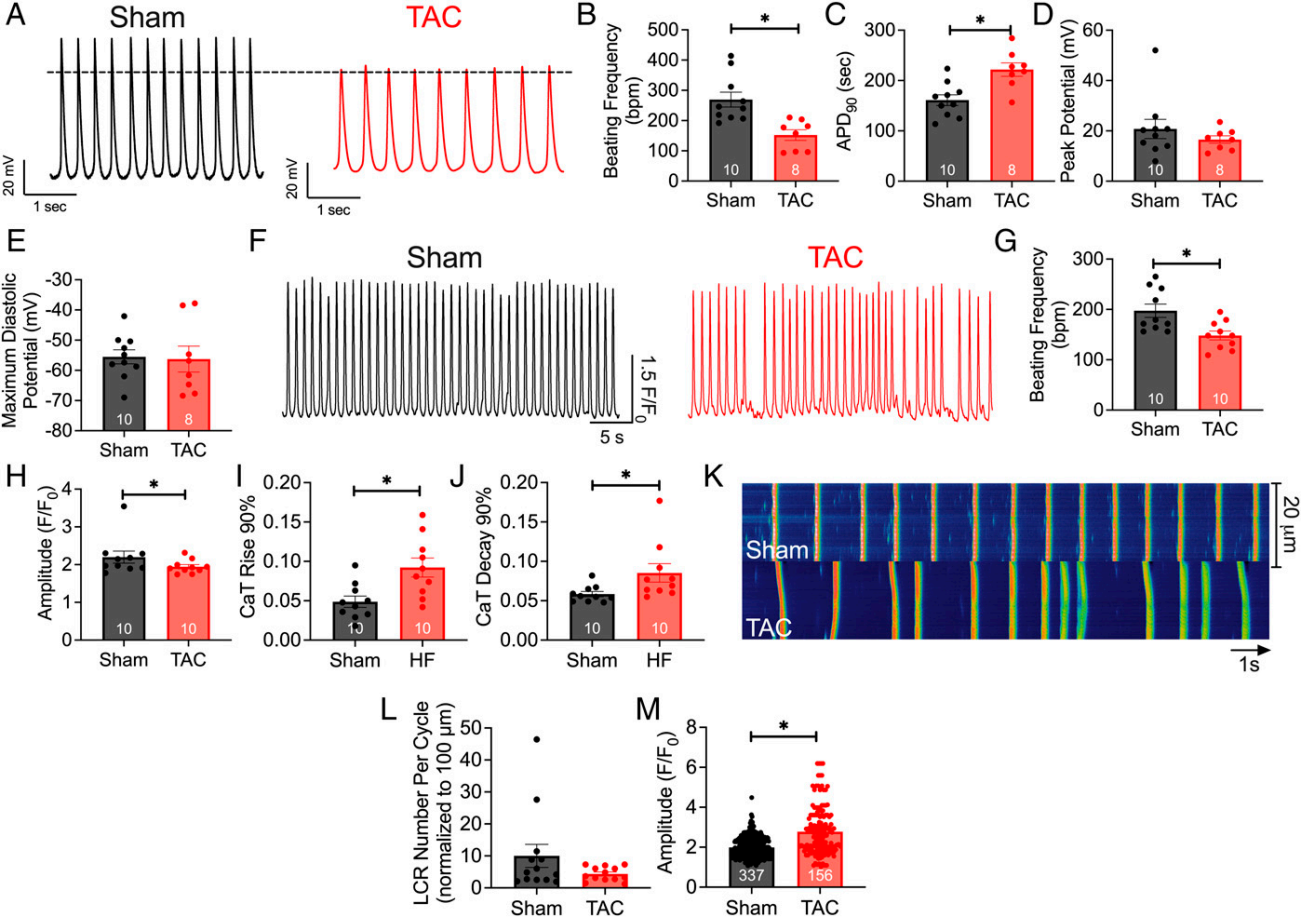
HF SANCs exhibited reduced frequency of action potentials and impaired Ca^2+^ transients and local Ca^2+^ release. (*A*) Representative AP recordings from sham and HF SANCs. (*B*) Summary data of beating frequency (bpm). (*C*–*E*) AP duration at 90% repolarization (*C*), maximum diastolic potential (*D*), and peak potentials (*E*). (*F*) Representative traces of Ca^2+^ transients from both groups are depicted. (*G*–*J*) Summary data of beating frequency (*G*), CaT amplitude (*H*), CaT rise at 90% maximum (*I*), and CaT decay from 90% maximum are shown (*J*). (*K*) Representative line scan images of Ca^2+^ transients and LCRs in diastole. (*L* and *M*) Summary data of numbers of LCRs per cycle normalized per 100 mm (*L*) and maximal amplitude of LCRs (*M*). Data are expressed as mean ± SEM. *P < 0.05. The numbers in the bar graphs represent the number of cells from three mice per group. Numbers in the bar graphs in *M* represent the total numbers of LCRs analyzed.

Since heart rates are influenced by intrinsic SAN function and autonomic nervous system input, we directly determined the spontaneous firing frequency from single isolated SANCs devoid of autonomic nervous system input. *SI Appendix*, Fig. S2 shows the landmark for SAN isolation. Representative AP tracings of sham and TAC SANCs are shown in Fig. 3*A*. Single SANCs isolated from HF showed a significant reduction in AP frequency (Fig. 3*B*) as well as a prolongation of the AP duration (APD) at 90% repolarization (APD_90_) (Fig. 3*C*; *P* < 0.05). There were no significant differences in maximum diastolic potential (Fig. 3*D*) or peak potential (Fig. 3*E*). Applications of carbonyl cyanide-*p*-trifluoromethoxyphenylhydrazone (FCCP), a mitochondrial uncoupler, resulted in a significant decrease in the firing frequency of APs in control SANCs, similar to APs in HF SANCs. This suggests a crucial role for mitochondria in SAN automaticity (*SI Appendix*, Figs. S2 and S3).

Ca^2+^ is an essential second messenger responsible for numerous cellular processes, including SAN automaticity. To determine changes in Ca^2+^ signaling in SANCs of HF mice, we recorded Ca^2+^ transients (CaTs) and the local Ca^2+^ release (LCR). The ryanodine receptors (RyR2) on the SR induce the local increase in Ca^2+^, and the summation of these local Ca^2+^ events triggers the whole-cell CaT (19). Representative images of CaTs for both groups are shown in Fig. 3*F*. In corroboration with our AP recordings, as well as the in vivo findings in HF (Fig. 2), TAC SANCs exhibited abnormal CaTs with irregular and reduced firing frequency (Fig. 3*G*; *P* < 0.05) and a reduction in CaT amplitudes relative to sham SANCs (Fig. 3*H*). Furthermore, CaT kinetics were significantly impaired, as demonstrated by a prolonged CaT rise (Fig. 3*I*) and lengthened CaT decay (Fig. 3*J*).

Additionally, LCRs, essential for Ca^2+^-mediated mitochondria–SR communication (20), were impaired in SANCs in HF. The frequency of LCR events per cycle, normalized to 100 μm, was reduced, as well as the amplitude, in TAC relative to sham SANCs (Fig. 3 *K*–*M*). Moreover, application of known mitochondrial toxins in control SANCs, rotenone (complex I inhibitor), antimycin A (complex III inhibitor), and oligomycin A (adenosine triphosphate [ATP] synthase inhibitor), decreased the CaT amplitude and frequency, as well as impaired LCR (*SI Appendix*, Figs. S4 and S5), similar to the findings in HF SANCs. Together, our data suggest that AP, CaT, and LCR are critically dependent on mitochondrial function and significantly impaired in HF SANCs.

### HF-Induced Morphological Changes to SAN Mitochondria.

To accurately assess the morphological alterations to SAN mitochondria, we isolated the SAN tissues according to Fenske et al. (21). Whole-mount SAN regions from sham and TAC mice were obtained and visualized using HCN4 as a marker of the SAN, as well as COX IV to label the inner mitochondrial membrane protein (*SI Appendix*, Fig. S2), demonstrating that our SAN tissue dissection consisted exclusively of the SAN tissue and revealing a dense network of mitochondria in SANCs. Western blot analyses showed similar levels of COX IV in SAN, atrial, and ventricular tissues (*SI Appendix*, Fig. S6).

**Fig. 4.**
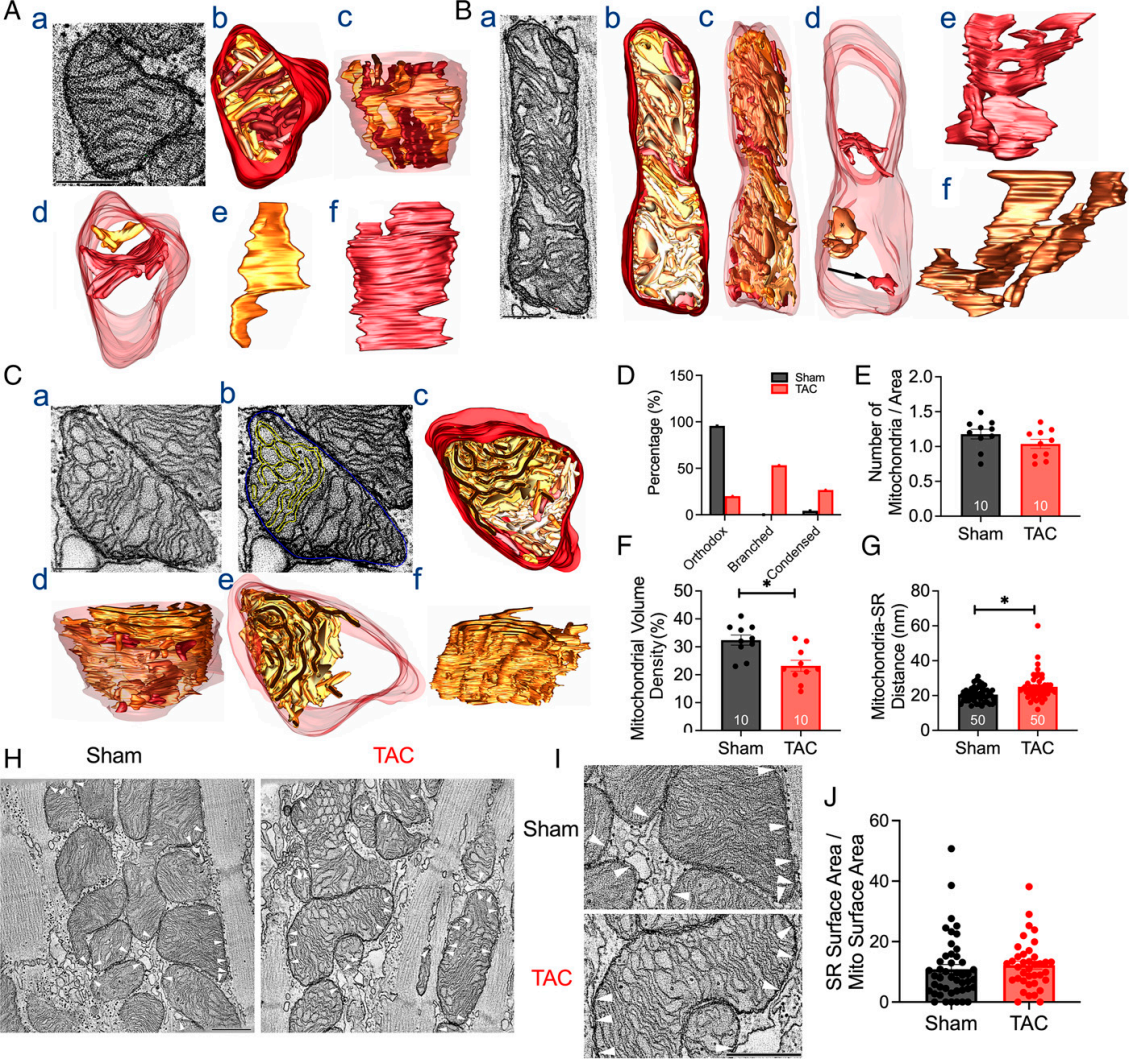
HF-induced morphological changes in SAN mitochondria. (*A*) Representative images of 3D reconstructions from EM tomography of a SAN mitochondrion from sham mice. Cristae are shown in various shades of brown, and the outer mitochondrial membrane (OMM) is shown in maroon. (*a*) The orthodox morphotype depicted here is a 1.6-nm-thick slice through the middle of a 400-nm-thick volume. (*b*) Top view of the surface-rendered volume of the mitochondrion after segmentation of the membranes. (*c*) Side view of the surface-rendered volume with the OMM made translucent. (*d*) Top view showing two representative cristae. (*e* and *f*) The two cristae are shown from the side. The second crista is wider and extends across the width of the mitochondrion for most of its height. (*B*) Representative images of 3D reconstructions of a SAN mitochondrion from TAC mice, showing a condensed mitochondrion. (*a*) The condensed type is shown here, with a 1.6-nm-thick slice through the middle of a 400-nm-thick volume. (*b*) Top view of the surface-rendered volume of the mitochondrion after segmentation of the membranes. (*c*) Side view of the surface-rendered volume of the OMM made translucent. (*d*) Top view showing three representative cristae. The top two are branched, with the lower of the two showing a portion of the top condensed (*). The arrow (*Bottom*) indicates the crista is a typically enlarged crista with no branching. (*e* and *f*) The top two cristae are shown from the side to emphasize their branching. (*C*) Representative images of 3D reconstructions of a SAN mitochondrion from TAC mice, showing a highly branched mitochondrion. (*a*) A 1.6-nm-thick slice through the middle of a 400-nm-thick volume shows the circular branching. (*b*) The same slice shows the extensive connectivity of a single crista (yellow). The OMM is in blue. (*c*) Top view of the surface-rendered volume of the mitochondrion after segmentation of the membranes. (*d*) Side view of the surface-rendered volume with the OMM made translucent. (*e*) The top view shows the highly branched crista to emphasize that it occupies roughly half of the mitochondrial volume. (*f*) Side view of the highly branched crista, which is seen to consist nearly entirely of connected lamella membranes. Scale bars in *A–C* represent 500 nm. Summary data of the percentage of mitochondria type (*D*), number of mitochondrial per area (*E*), mitochondrial volume (*F*), and mitochondria–SR distance are shown (*G*). (*H*) Representative EM images showing mitochondria–SR contact sites (white arrowheads) from sham and HF SANCs. (*I*) Enlarged portions of the EM images from *H*. (Scale bars, 500 nm [*H* and *I*].) (*J*) Summary data for SR contact surface area/mitochondria surface area for sham and HF SANCs (*n* = 46 and 37 mitochondria for sham and HF SANCs, respectively). Data are expressed as mean ± SEM. *P < 0.05.

To visualize and quantify the ultrastructure of the mitochondria (*SI Appendix*, Table S2), and acquire three-dimensional (3D) reconstructions of the mitochondria, we utilized TEM and EM tomography, respectively. EM tomography revealed a significant shift in the types of mitochondria, as evidenced by representative 3D reconstructions of the mitochondria from the SAN of sham (Fig. 4*A*) and HF mice (Fig. 4 *B* and *C*). Classically, normal mitochondria are divided into two classes—orthodox and condensed (22). The orthodox morphotype was typically seen in situ in sham SAN tissues, as depicted in the 3D reconstructions (Fig. 4*A*). In contrast, SAN mitochondria from TAC mice exhibited a more condensed morphotype (Fig. 4*B*) or a highly branched morphotype (Fig. 4*C*). The morphotype breakdown of SAN mitochondria from sham animals is 96% orthodox and 4% condensed, while the morphotype breakdown of SAN mitochondria from HF animals is 20% orthodox with no branching, 27% condensed with no branching, and 53% branched (Fig. 4*D*). SAN mitochondria from TAC mice exhibited a relatively high proportion of mitochondria with branched cristae, absent in SAN mitochondria from sham mice. The number of mitochondria per area was not different between the two groups (Fig. 4*E*). However, SAN mitochondria from TAC mice exhibited lower mitochondrial volume than SAN mitochondria from sham mice (Fig. 4*F*). Notably, there was a significant increase in mitochondria–SR distance (Fig. 4*G*). Our data support the significant structural remodeling of mitochondria in HF, with increased the condensed or branched forms in the HF SAN. Our data also suggest a disruption of the mitochondria–SR microdomains, as evidenced by the increased mitochondria–SR distance.

Fig. 4 *H* and *I* illustrates representative examples of mitochondria–SR contacts (white arrowheads). We directly quantified SR contact surface area/mitochondria surface area and found no significant differences in the SR contact surface area between sham and HF SANCs. Specifically, the SR contact surface area/mitochondria surface area was 10.9 ± 10.4% (mean ± SD, *n* = 46 mitochondria) for sham and 12.2 ± 8.0% (mean ± SD, *n* = 37 mitochondria) for HF SANCs.

Similar findings were demonstrated using TEM showing a significant decrease in the mitochondrial area (*SI Appendix*, Fig. S7*A*; *P* < 0.0001) and perimeter (*SI Appendix*, Fig. S7*B*; *P* < 0.0001) in HF SANCs. Even though the aspect ratio, or the length-to-width ratio, was not different (*SI Appendix*, Fig. S7*C*), measurement of skewness, or asymmetry in distribution, indicated a significant increase in smaller mitochondria in the HF group (*SI Appendix*, Fig. S7*D*; *P* < 0.001). Feret’s diameter, computed as the longest distance between two points on the mitochondria, was significantly lower in the TAC group (*SI Appendix*, Fig. S7*E*; *P* < 0.0001). Additionally, SAN mitochondria from TAC mice showed a significant deviation away from circularity (*SI Appendix*, Fig. S7*F*; *P* < 0.05). The plotted relative frequency distribution in *SI Appendix*, Fig. S7*A* demonstrated smaller mitochondria (area < 0.2 μm^2^, ∼25% of total mitochondria) in the TAC group versus the sham group (∼10%). Consistent with EM tomography showing lower mitochondrial volume, TEM data suggest significantly smaller mitochondria in the HF SAN.

### HF-Reduced Mitochondria and SR Colocalization.

**Fig. 5.**
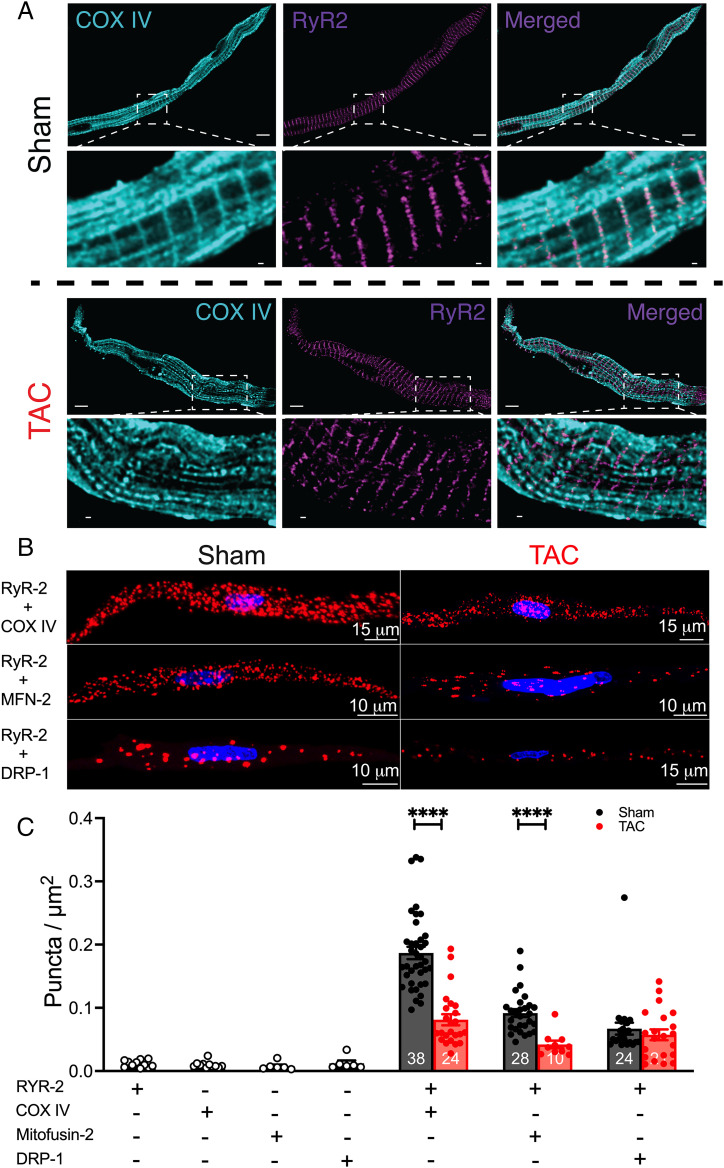
HF reduced mitochondria and SR colocalization and disrupted mitochondria–SR microdomains. (*A*) Representative stimulated emission depletion microscopy images of sham and HF SANCs colabeled with RyR2 (purple) and COX IV (cyan). Two SANCs were seen in the sham group. (Scale bars, 5 μm [*Upper*] and 0.5 μm [enlarged images, *Lower*] for each group.) (*B*) PLA showing representative immunofluorescence confocal microscopy 3D rendered images, with PLA puncta (red) and DAPI (blue) from sham and HF SANCs, labeled with RyR2 + COX IV, RyR2 + Mfn2, and RyR2 + DRP-1. Positive cross-reactivity, which reflects an intermolecular distance of 40 nm or less, is detected as puncta, and the nuclei are depicted in blue. (*C*) Summary data of PLA fluorescent puncta per cell area (puncta per square micrometer) for the different combinations, including negative controls with one primary antibody. Data are represented as mean ± SEM. ****P < 0.0001. The symbols represent the number of cells within the bar graphs (*n* = 3 mice for each group).

We further tested the spatial proximity between the SAN mitochondria and the SR since the close distance between the two organelles is crucial for proper cross-talk. High-resolution immunofluorescence imaging of SANCs using anti-RyR2 and –COX IV antibodies demonstrated colocalization with similar spatial organization between SR and mitochondria under sham conditions (Fig. 5*A*). However, no distinct colocalization between RyR2 and COX IV was observed in HF SANCs (Fig. 5 *A*, *Lower*). MitoTracker Deep Red FM and tetramethylrhodamine methyl ester (TMRM), both mitochondria-targeted dyes, show similar mitochondrial distribution (*SI Appendix*, Fig. S8) to COX IV staining.

We then used the proximity ligation assay (PLA) as an additional analytical tool to examine the association between RyR2 and COX IV, Mfn2, or dynamin-related protein 1 (DRP-1). PLA fluorescence puncta are only observed if proteins of interest are ≤40 nm apart. Negative controls were performed, showing the absence of PLA signal when at least one primary antibody was omitted (*SI Appendix*, Fig. S9). Robust fluorescent puncta were detected in SANCs isolated from sham animals colabeled for RyR2 and COX IV, Mfn2, or DRP-1 but not in TAC SANCs (Fig. 5*B*). Summary data in Fig. 5*C* demonstrate a significant decrease in fluorescent puncta in TAC SANCs, colabeled with RyR2 and COX IV, or Mfn2 compared with sham (*P* < 0.0001). The results suggest that mitochondria in SANCs are located within nanometer proximity (<∼40 nm) to the SR. However, the spatial proximity between the mitochondria and SR in HF conditions was significantly disrupted, consistent with findings from EM tomography showing a significant increase in mitochondria–SR distance (Fig. 4*G*).

### Mitochondrial Function Was Impaired in HF SANCs.

**Fig. 6.**
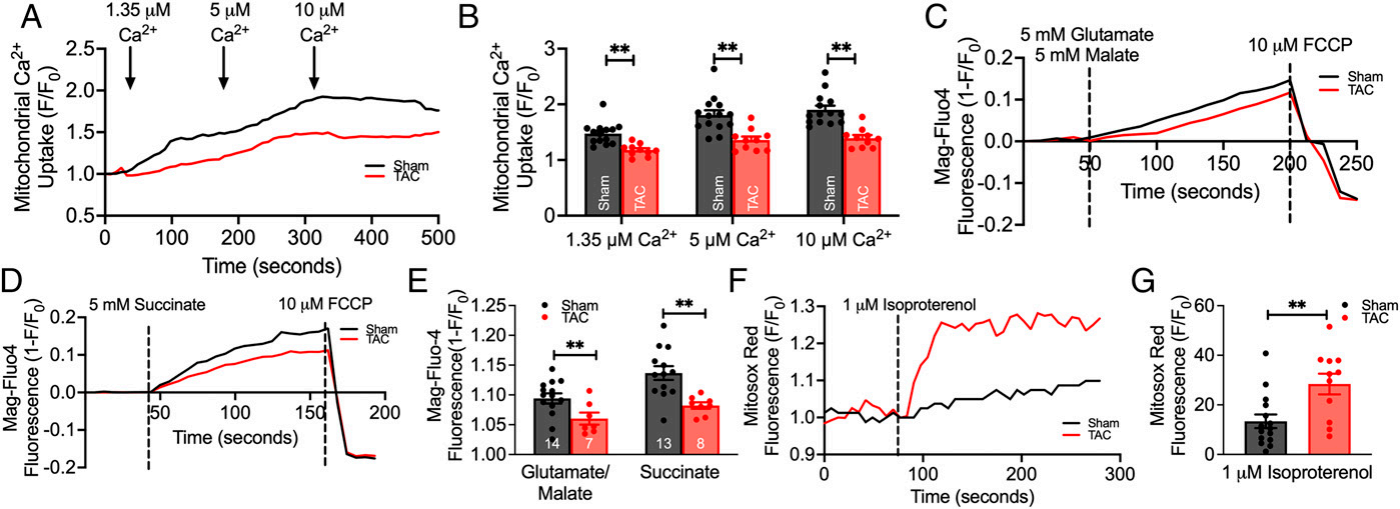
Mitochondrial function was impaired in HF SANCs. (*A*) Representative traces of mitochondrial Ca^2+^ uptake (monitored by X-Rhod-1 fluorescence) from sham compared with HF SANCs, challenged with increasing extramitochondrial [Ca^2+^]. (*B*) Summary data of the normalized fluorescence intensity after perfusion of various Ca^2+^ concentrations are shown (*n* = 14 sham and *n* = 10 TAC SANCs). (*C* and *D*) Production of ATP was monitored using Mag-Fluo-4 after the perfusion with complex I (glutamate/malate; *n* = 14 sham and *n* = 10 TAC SANCs) (*C*) and complex II (succinate; *n* = 13 sham and *n* = 8 TAC SANCs) (*D*) substrates, as shown by the representative traces. (*E*) Summary data of Mag-Fluo-4 intensity after substrate application are displayed. (*F*) Measurement of ROS production was monitored using MitoSox red, as shown by the representative traces. (*G*) Summary data of ROS production after isoproterenol perfusion are shown (*n* = 15 sham and *n* = 11 TAC SANCs). Data are expressed as mean ± SEM. **P < 0.01.

Mitochondria are responsible for many vital cellular processes, and their normal function is crucial for cell survival. To assess changes in mitochondrial function, we examined Ca^2+^ uptake, ATP production, and ROS production. There was a significant decrease in mitochondrial Ca^2+^ uptake (Fig. 6 *A* and *B*) when permeabilized TAC SANCs were challenged with increasing extramitochondrial concentrations of Ca^2+^ (1.35, 5, and 10 μM), relative to SANCs from sham mice, as indicated by the lowered response in X-Rhod-1 fluorescence intensity (*P* < 0.01; Fig. 6*B*). Additionally, when the SANCs from TAC mice were supplied with complex I substrates (5 mM glutamate/5 mM malate; Fig. 6 *C* and *E*) and complex II substrate (5 mM succinate; Fig. 6 *D* and *E*), ATP level was significantly lower in SANCs from TAC mice, as indicated by the level of Mg-Fluo-4 intensity (*P* < 0.01).

ATP-sensitive K^+^ currents (*I*_K,ATP_s) have been shown to contribute to the spontaneous AP firing in the SAN (23). To test whether the decrease in ATP production may affect spontaneous beating frequency via the ATP-sensitive K^+^ channels (K_ATP_s), we recorded APs in SANCs before and after glibenclamide, a known K_ATP_ channel inhibitor. Glibenclamide (10 μM) increased AP firing frequency as well as decreased maximum diastolic potentials (MDPs) and peak potentials in both sham and HF SANCs (*SI Appendix*, Fig. S10). Moreover, both groups exhibited a similar increase in AP firing frequency after glibenclamide application (*SI Appendix*, Fig. S10*E*), suggesting that bradycardia and SAN dysfunction are not directly attributable to *I*_K,ATP_ activation in HF.

Furthermore, SANCs from TAC mice exhibited an elevated production of superoxide, predominantly ROS, quantified by the increased level of MitoSox red fluorescence after perfusion with isoproterenol, a β-adrenergic receptor (β-AR) agonist (Fig. 6 *F* and *G*). Pretreatment of HF SANCs with 1 μM MitoTempo, a known mitochondrial antioxidant (24), restored the CaT frequency (*SI Appendix*, Fig. S11), suggesting that oxidative stress contributes, at least in part, to the abnormal Ca^2+^ dynamics in HF SANCs. Together, the data suggest that disruptions in mitochondrial structure and mitochondria–SR connectomics translate into a significant impairment in mitochondrial function in the HF SAN.

### SAN-Specific *Mfn2* Knockdown Mice Exhibited SAN Dysfunction.

**Fig. 7.**
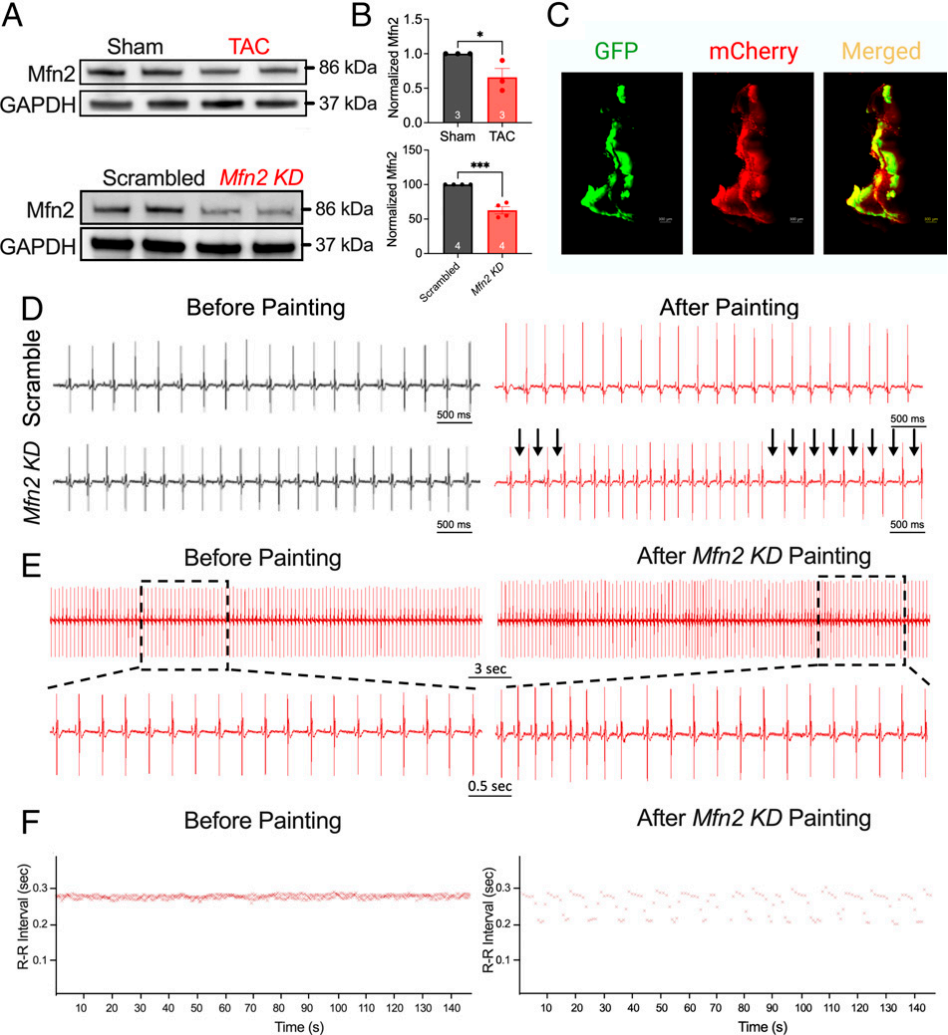
SAN-specific *Mfn* KD mice exhibited SAN dysfunction. (*A*) Representative relative expression of Mfn2 in the SAN from sham and TAC mice and from scrambled and *Mfn2* KD mice. GAPDH served as a loading control and was used to normalize the expression of Mfn2. (*B*) Summary data of normalized Mfn2 expression are displayed. (*C*) Representative images of GFP, mCherry, and merged signal of a SAN tissue, painted with an Mfn2 construct, from a GCaMP mouse. ECG recordings were then acquired from scrambled and Mfn2 painted mice. (*D*) Representative ECG traces from scrambled sequence compared with CRISPR-Cas9-*Mfn2*–mediated KD mice before (*Left*) and after (*Right*) SAN-specific painting, showing RR-I prolongation (arrows). (*E*) A closer examination of an *Mfn2* KD mouse before and after painting surgery. (*F*) A representative RR-I over time. *n* = 6 for each group. Data are expressed as mean ± SEM. *P < 0.05, ***P < 0.001.

To determine the possible underlying mechanisms for the observed disruption in mitochondria–SR connectomics, we quantified the expression of Mfn2, a primary protein that tethers the mitochondria to the SR, forming microdomains conducive to interorganelle communication (25, 26). Mfn2 protein expression was significantly decreased in SAN tissues from TAC relative to sham mice (Fig. 7 *A* and *B*).

To test the critical roles of Mfn2 in the integrity of mitochondria–SR microdomains, we generated SAN-specific CRISPR-Cas9–mediated knockdown (KD) of *Mfn2*. We delivered liposomes containing either scrambled single-guide RNAs (control sgRNAs) or sgRNAs targeting the *Mfn2* gene (pNV-sgRNA-Cas9-2A-mCherry; *SI Appendix*, Fig. S12) to the epicardial surface of the SAN region in GCaMP8 mice (27), using the previously described painting technique (28). Since the transgenic GCaMP8 mice expressed a Ca^2+^ biosensor under the control of the HCN4 promoter, we documented the success of the painting method by the overlap of the green fluorescent protein (GFP) and mCherry signal from the CRISPR-Cas9 constructs (Fig. 7*C*). Using this technique, we significantly reduced the protein expression level of Mfn2 in SAN tissues as assessed by Western blot analyses (Fig. 7 *A* and *B*). CRISPR-Cas9-*Mfn2* KD mice experienced SAN dysfunction (Fig. 7 *D* and *E*) without compromised cardiac function (*SI Appendix*, Fig. S13). Surface ECG recordings from *Mfn2* KD mice exhibited periods of RR prolongation, similar to ECG recordings from TAC mice and absent in the scrambled (control) mice (Fig. 7*D*). ECG traces after treatment with the *Mfn2* targeting vector demonstrated multiple periods of RR-I prolongation (Fig. 7*E*). Indeed, these mice exhibited pronounced HRV relative to baseline (Fig. 7*F*).

### β-AR–Induced cAMP Signals at the SR Were Impaired in HF and *Mfn2* KD SANCs.

**Fig. 8.**
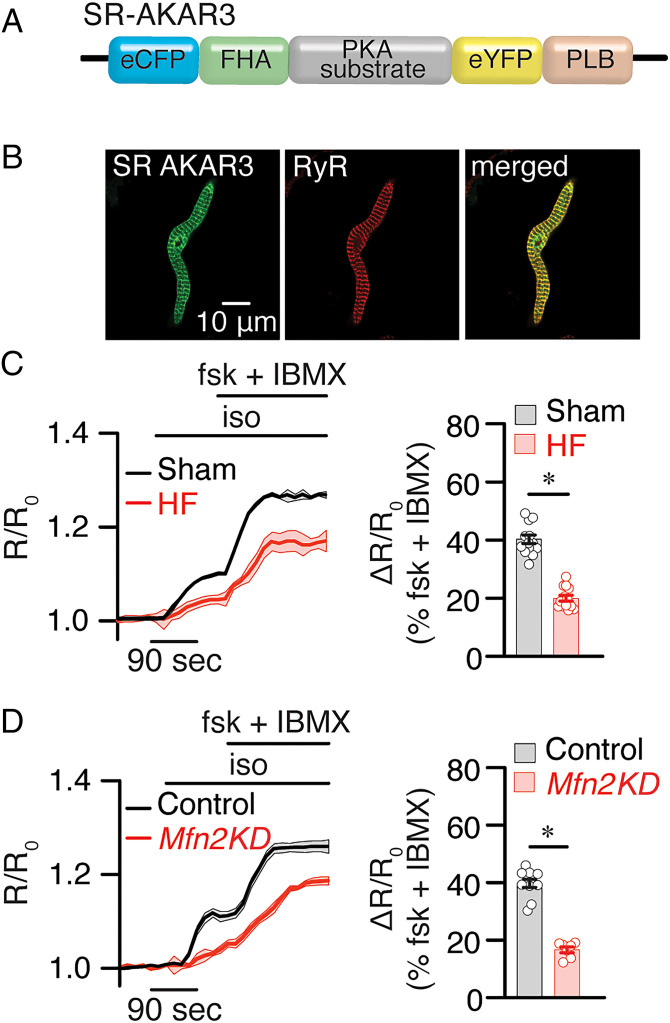
β-AR–induced cAMP signals at SR and OMM compartments were impaired in HF and *Mfn2* KD SANCs. (*A* and *B*) Schematic of the SR-targeted FRET-based PKA reporter. Confocal images of SANs expressing SR-AKAR3 show the localization of the biosensor. (*C* and *D*) Time course of changes in the magnitude of normalized FRET responses (*R*/*R*_0_) in SANCs expressing SR-AKAR3 and OMM-AKAR3 upon application of β-AR agonist isoproterenol (iso, 100 nM) in the presence of the adenylyl cyclase activator forskolin (fsk) and phosphodiesterase inhibitor IBMX in sham and HF cells (*C*) or control and *Mfn2* KD SANCs (*D*). Bar graphs that represent the maximal increases in the FRET ratio of these sensors are plotted. *n* ≥ 5 cells from three preparations per condition. Data represent the mean ± SEM. *P < 0.05 by Kruskal–Wallis with Dunn’s multiple comparisons.

Emerging evidence suggests that localized cAMP signaling occurs at mitochondria–SR microdomains (29), with multiple signaling pathways converging in these regions. Therefore, disruption of the mitochondria–SR microdomains observed in HF may substantially affect not only Ca^2+^ but also cAMP and protein kinase A (PKA) signaling. To directly test the possible alterations in cAMP/PKA signaling, we use a fluorescence resonance energy transfer (FRET)–based biosensor that reports PKA activity specifically in the SR region (SR-AKAR3) (30). The sensor was transduced in SANCs as we have previously described (31) from sham, TAC, and SAN-specific *Mfn2* KD mice (Fig. 8 *A* and *B*). High-resolution immunofluorescence images demonstrating the specific localization of the biosensor are shown (Fig. 8*B*). FRET signals at the SR were significantly reduced in TAC SANCs relative to sham SANCs after β-AR stimulation with isoproterenol and in response to forskolin and 3-isobutyl-1-methylxanthine (IBMX) (Fig. 8*C*). Similar findings were observed in SAN-specific *Mfn2* KD compared with control (treated with scrambled sgRNA, Fig. 8*D*). Our data suggest that the disruption of mitochondria–SR microdomains seen in HF and SAN-specific *Mfn2* KD impaired localized SR PKA activity and likely cAMP signaling.

## Discussion

The SAN is the primary pacemaking region, generating spontaneous APs that propagate throughout the heart. However, in HF, its automaticity is impaired, contributing to increased morbidity and mortality in HF patients (2, 3, 32). Various components of the membrane and Ca^2+^ clocks (33) are shown to be significantly remodeled during HF. However, the roles of organelle connectomics in SAN function remain unknown. We took advantage of a preclinical model of HF and directly demonstrate SAN dysfunction in vivo and in vitro in isolated SANCs, evidenced by significant bradyarrhythmia and a significant reduction in AP firing frequency, respectively (Figs. 2 and 3). Remarkably, considerable remodeling of the mitochondria with disruption of the mitochondria–SR microdomains is demonstrated in SANCs in HF mice using high-resolution imaging and EM tomography (Figs. 4 and 5). Disruption of the mitochondria–SR microdomains critically impacts mitochondrial function and energy production (Fig. 6). Mechanistically, the expression of Mfn2, an essential protein involved in tethering the mitochondria and SR, is significantly reduced in HF SAN. SAN-specific gene silencing of *Mfn2* results in SAN dysfunction with disruption of local PKA activity and likely cAMP signaling (Figs. 7 and 8). The current study supports the critical roles of mitochondria–SR connectomics in SAN automaticity.

### Previous Studies on SAN Dysfunction in HF.

In patients with congestive HF, the intrinsic cycle length increased with prolongation of sinoatrial conduction time (3). Previous studies have documented significant alterations of the membrane and Ca^2+^ clocks in HF SANs (33). In a rapid-pacing canine model of HF, the enhanced late diastolic Ca^2+^ was significantly impaired in the superior SAN. Ectopic pacemaking activity occurred in the lower crista terminalis in the presence and absence of β-adrenergic stimulation (6). Moreover, expression of the HCN channel at both messenger RNA and protein levels decreased in a similar model of HF (8). With a volume and pressure overload rabbit HF model, there was a significant increase in the in vitro and in vivo intrinsic cycle length of the SAN (34). Verkerk et al. also found an increase in intrinsic cycle length, with a concomitant decrease in the diastolic depolarization rate (9). There was a decline in the pacemaker current without alterations in T- and L-type Ca^2+^ currents.

### Mitochondria Are the Fuel Source for SAN Automaticity.

The heart requires substantial energy to sustain itself on a beat-to-beat basis. Normal-functioning mitochondria are crucial to cardiac cell survival, and dysfunctional mitochondria have been associated with several cardiovascular diseases, including ischemia-reperfusion injury, hypertension, diabetic cardiomyopathy, cardiac hypertrophy, and HF (35). Pacemaking cardiomyocytes are endowed with a dense mitochondrial network that supplies the bulk energy through oxidative phosphorylation (10, 36). Although mitochondrial dysfunction in HF has been well-studied in ventricular cardiomyocytes, where the energy starvation hypothesis was first proposed (37), there is a significant gap in our knowledge of mitochondria’s roles in the HF SAN. Since SANCs are noncontractile, autorhythmic cells with a high density of mitochondria (10) (Fig. 4 and *SI Appendix*, Figs. S2 and S6), alterations in mitochondria or communications with the mitochondria may contribute to SAN dysfunction commonly seen with HF (3).

Mitochondria play critical roles in the SAN’s automaticity through their Ca^2+^ handling and energy production. The spontaneous depolarization of the SAN triggers the influx of Ca^2+^ through the L-type Ca^2+^ channels, leading to SR Ca^2+^ release through RyR2 (10, 38). SR Ca^2+^ release generates the beat-to-beat rise of intracellular Ca^2+^ (Ca^2+^_i_) within microdomains between the mitochondria and SR, critical for localized signaling (39, 40). Mitochondria and SR connectomics are obligatory for coupling energy production with the cell’s metabolic demands, mediated in part by mitochondrial Ca^2+^ (10, 11, 39). Ca^2+^ within the mitochondrial matrix stimulates mitochondrial enzymes (41), producing reduced equivalents that can be oxidized for energy production (42).

An increase in intracellular Ca^2+^ within SANCs is extruded via the plasma membrane Na^+^/Ca^2+^ exchanger, generating a net inward current that contributes to the spontaneous depolarization, termed the Ca^2+^ clock (5). The periodic rise and fall of Ca^2+^ require a beat-to-beat reestablishment of the ionic gradient, fueled primarily by fatty acid oxidation in cardiomyocytes (43). With the influx of Ca^2+^, and the rise in Ca^2+^ through Ca^2+^-induced Ca^2+^ release, Ca^2+^ acts as a second messenger to match energy production with energy demand, which is especially important during increased sympathetic tone where energy demand is elevated. Thus, mitochondria participate in the SAN’s automaticity by coupling metabolic supply with demand. As directly demonstrated in this study, application of known mitochondrial toxins to isolated SANCs in vitro resulted in a significant impairment in AP frequency, CaT, and LCR (*SI Appendix*, Figs. S3–S5), recapitulating the findings observed in isolated SANCs from HF mice (Fig. 3). These results suggest that functional mitochondria are essential for SAN automaticity.

### Ultrastructural Changes in Mitochondria in the HF SAN.

We observed an accumulation of damaged mitochondria in the HF SAN (Fig. 4) with a reduced ability to uptake Ca^2+^ at increasing extramitochondrial [Ca^2+^], contributing to a reduction in energy generation when the mitochondria were supplied with either complex I (glutamate/malate) or complex II (succinate) substrates. Indeed, EM tomography revealed substantial structural remodeling of the mitochondria in the HF SAN, transitioning more orthodox mitochondria to more condensed and highly branched mitochondria. Condensed mitochondria in situ may indicate two functional correlates that are not mutually exclusive—ramped-up ATP production and response to stress (44). The rate of ATP production can be significantly higher in condensed mitochondria compared with orthodox mitochondria, and usually stems from signals indicating higher local energy demands. However, our findings demonstrated that complex I– and complex II–mediated energy production are significantly decreased in HF SANCs (Fig. 6), suggesting that although the morphology of the mitochondria is crucial, communication with them may be physiologically necessary to enhance energy generation.

### Altered Mitochondria–SR Microdomains in the HF SAN.

Microdomains between the mitochondria and the SR serve critical roles in spatially localized signaling, enabling proper communication (45). Mfn2, a mitochondrial fusion protein, tethers the mitochondria with the SR (14, 46). Ablation of *Mfn2* produced mitochondrial fragmentation and impaired cardiac function (47). Moreover, *Mfn2* deficiency reduces the mitochondria–SR contact length (increased distance between the two organelles) and decreases mitochondrial Ca^2+^ uptake in ventricular myocytes (48). With the disruption of the microdomains, the bioenergetic feedback from Ca^2+^-induced stimulation is diminished, while the feedback from ROS, cAMP, and other messengers is impaired. Mutations in the *Mfn2* gene have been linked to Charcot–Marie–Tooth syndrome, and dysregulation of *Mfn2* may be linked to multiple diseases, including Alzheimer’s and Parkinson’s diseases (49).

In the present study, we demonstrate that the mitochondria and SR are localized in close proximity (<40 nm) in isolated SANCs from sham-operated animals by probing for Mfn2 and COX IV (inner mitochondrial membrane protein) and RyR2 colocalization. In contrast, HF SANCs show a significant increase in the spatial proximity between the SR and mitochondria, suggesting structural remodeling within the mitochondria and at the subcellular level with the disruption of their microdomains (Fig. 5). Further definitive evidence is supported by the EM tomography revealing an increased distance between the mitochondria and SR in HF SANCs (Fig. 4). Mechanistically, there is a significant decrease in Mfn2 expression, suggesting that Mfn2 may partly mediate the impairment in mitochondria–SR microdomains.

Importantly, SAN-specific *Mfn2* KD supports the mitochondria–SR communication’s significance in regulating SAN automaticity. SAN-specific *Mfn2* KD mice exhibit SAN dysfunction, with sinus bradyarrhythmia (Fig. 7) evidenced in HF (Fig. 2). With the disruption of the mitochondria–SR microdomains in the *Mfn2* KD SAN, cAMP signaling is significantly impaired, with similar findings in HF SANs (Fig. 8). Indeed, mitochondria–SR communications play crucial roles in localized cAMP signaling. Like Ca^2+^, cAMP significantly impacts oxidative phosphorylation, apoptosis, and mitochondrial dynamics (50). These microdomains have been shown to be communication hubs for the complex interactions between different second messengers, such as Ca^2+^ and cAMP.

### Conclusions.

The current study demonstrates that SAN dysfunction occurs concurrently with HF development in a preclinical murine model with abnormal automaticity in isolated SANCs. Structural remodeling of mitochondria and their microdomains occur in HF, with a shift in mitochondrial morphotype and disruption of the mitochondria–SR microdomains. Functionally, this translated to depressed mitochondrial energy production, reduced mitochondrial Ca^2+^ uptake, impaired cAMP signaling, and increased ROS generation. SANCs challenged with mitochondrial toxins exhibit significant impairment in LCR, CaT, and firing frequency, suggesting that functionally impaired mitochondria impact beating frequency. Mechanistically, Mfn2 expression is decreased in HF, and SAN-specific knockdown of *Mfn2* recapitulates SAN dysfunction and decreased localized cAMP signaling. The findings support the critical roles of mitochondria–SR connectomics in SAN dysfunction commonly seen with HF.

## Materials and Methods

Details are provided in *SI Appendix*, *Materials and Methods*.

Animal studies were performed following approved protocols of the Institutional Animal Care and Use Committee at the University of California, Davis, and adhere to the guidelines published by the NIH (51). Ten- to 16-wk-old male and female WT (C57BL/6J) and GCaMP8 C57BL/6J mice were used for this study.

### TAC Surgery.

WT mice were randomized to undergo the sham or TAC surgeries and were followed for 8 wk. TAC surgery was performed as previously described (18). Briefly, the transverse aorta was visualized and ligated to the size of a 27-gauge needle. The sham procedure was identical, except for the ligation.

### SAN-Specific CRISPR-Cas9–Mediated *Mfn2* KD.

We delivered liposomes containing either scrambled single-guide RNAs (control sgRNAs) or sgRNAs targeting the *Mfn2* gene (pNV-sgRNA-Cas9-2A-mCherry; *SI Appendix*, Fig. S12) to the epicardial surface of the SAN region in GCaMP8 mice (27), using the previously described painting technique (28).

### Statistical Analysis.

All data are reported as mean ± SE unless otherwise stated. Statistical significance was determined using the Student paired *t* test. A value of *P* < 0.05 was considered statistically significant.

## Supplementary Material

Supplementary File

## Data Availability

All study data are included in the article and/or *SI Appendix*.
