# Supplementary material for: Disruption of mitochondria–sarcoplasmic reticulum microdomain connectomics contributes to sinus node dysfunction in heart failure
Source: Proc Natl Acad Sci U S A. 2022 Aug 31;119(36):e2206708119. doi: 10.1073/pnas.2206708119 (PMC9456763; doi:10.1073/pnas.2206708119)
Supplement: Supplementary File [file pnas.2206708119.sapp.pdf]

SUPPORTING INFORMATION

Disruption of Mitochondria-Sarcoplasmic Reticulum Microdomain Connectomics Contributes to Sinus Node Dysfunction in Heart Failure

Lu Ren[1], Raghavender R. Gopireddy[2], Guy Perkins[3], Hao Zhang[4], Valeriy Timofeyev[1], Yankun Lyu[1], Daphne A. Diloretto[1], Pauline Trinh[1], Padmini Sirish[1], James L. Overton[1], Wilson Xu[1], Nathan Grainger[5], Yang K. Xiang[2], Elena N. Dedkova[6], Xiao-Dong Zhang[1], Ebenezer N. Yamoah[7], Manuel F. Navedo[2], Phung N. Thai[1,7,*], Nipavan Chiamvimonvat[1,2,8*]

[1]Department of Internal Medicine, Division of Cardiovascular Medicine, UC Davis
[2]Department of Pharmacology, UC Davis
[3]National Center for Microscopy and Imaging Research, UCSD
[4]Cardiovascular Institute, Stanford University
[5]Department of Physiology and Membrane Biology, UC Davis
[6]Department of Molecular Biosciences, School of Veterinary Medicine, UC Davis
[7]Department of Physiology and Cell Biology, University of Nevada, Reno
[8]Department of Veterans Affairs, Northern California Health Care System, Mather, CA

* Denotes co-corresponding authors

Corresponding Authors:
Phung N. Thai, Ph.D. and Nipavan Chiamvimonvat, M.D.
Department of Internal Medicine, Division of Cardiovascular Medicine
University of California, Davis
451 Health Science Drive, GBSF 6315
Davis, CA 95616
Department of Veterans Affairs, Northern California Health Care System
10535 Hospital Way Mather, CA 95655
E-mails: pnthai@ucdavis.edu; nchiamvimonvat@ucdavis.edu

This PDF file includes:
Supporting text
Figures S1 to S13
Tables S1 to S2
SI References

DETAILED MATERIALS AND METHODS

Animal studies were performed following approved protocols of the Institutional Animal Care and Use Committee at the University of California, Davis, and adhere to the guidelines published by the US National Institutes of Health (8th Edition). 10-16 week-old male and female wild-type (WT, C57BL/6J) and GCaMP8 (1, 2) in C57BL/6J background were used for this study.

Transverse Aortic Constriction (TAC) Surgery

WT mice were randomized to undergo the sham or transverse aortic constriction (TAC) surgeries and were followed for 8 weeks. TAC surgery was performed as previously described (3, 4). Briefly, the transverse aorta was visualized and ligated to the size of a 27-gauge needle. The sham procedure was identical, except for the ligation.

SAN-Specific CRISPR/Cas9-Mediated Gene Silencing of *Mfn2*

To test the critical roles of Mfn2 in the integrity of mitochondria-SR microdomains, we generated SAN-specific CRISPR/Cas9-mediated gene silencing (5-7) of *Mfn2*. We delivered liposomes containing either scrambled single-guide RNAs (control sgRNAs) or sgRNAs targeting the *Mfn2* gene (pNV-sgRNA-Cas9-2A-mCherry, **Supplementary Figure 9**) to the epicardial surface of the SAN region in GCaMP8 mice, using the previously described painting technique (8-10).

Echocardiography

We used the VisualSonics Vevo 2100 ultrasound system with the MS 550D probe (FUJIFILM VisualSonics, Inc., Toronto, Canada) to perform echocardiography as we have previously described (4, 11, 12). Pulse wave Doppler was performed at the left and right common carotids after the first week to determine successful ligation of the aorta. Systolic function (B-

mode videos and M-mode images) was quantified in conscious mice, and diastolic function (tissue Doppler and pulse-wave Doppler) was measured under anesthesia using 0.5-1% isoflurane.

Electrocardiography

Sham and HF mice underwent ECG telemetry recordings. Transmitters (Data Sciences International, St. Paul, MN) were implanted in mice to record ECGs as described (13). After the surgical procedure, mice were allowed to recover for at least 4 days before baseline 24-hour recordings commenced. The following 24-hour recordings were performed at 8 weeks when TAC mice developed evidence of HF, as verified by echocardiography.

Control (Scrambled) and *Mfn2 KD* mice underwent surface ECG recordings as previously described (14). Mice were injected with ketamine/xylazine (80 mg/kg / 5 mg/kg, i.p.) ten minutes prior to ECG recordings.

Fibrosis Assessment

After 8 weeks, hearts were harvested, fixed in 4% paraformaldehyde solution in phosphate-buffered saline (PBS), and embedded in paraffin. Five-micrometer coronal sections were acquired. Sections were deparaffinized in a series of xylene/alcohol(3). The sections were stained for hematoxylin and eosin (H&E), Picrosirius Red (PSR), and Masson's Trichrome (MT). Fibrosis quantification was performed in a blinded manner using NIH ImageJ, and determined as the percentage of the collagen area (red for PSR and blue for MT) per total area of the left ventricle.

SAN Cell (SANC) Isolation

SANCs were isolated as described previously (15-17). Mice were anesthetized by intraperitoneal injection of 80 mg/kg of ketamine and 5 mg/kg of xylazine. The heart was excised and placed into Tyrode's solution (35°C) containing (in mM): 140 NaCl, 5.0 HEPES, 5.5 Glucose, 5.4 KCl, 1.8 $CaCl_2$, and 1.0 $MgCl_2$ (pH 7.4). The SAN tissue was dissected with a dissecting scope

according to the landmarks of the heart defined by the orifice of superior vena cava, crista terminalis, and atrial septum. SAN tissue was digested in low Ca^{2+} solution (pH 6.9), containing collagenase B (0.54 U/mL, Sigma-Aldrich, Burlington, MA), elastase (18.9 U/mL, Sigma), and protease type XIV (1.79 U/mL, Sigma) for 30 min at 37°C. After digestion, the tissue was washed with Kraft-Bruhe medium containing (in mM): 100 potassium glutamate, 5 HEPES, 20 glucose, 25 KCl, 10 potassium aspartate, 2 $MgSO_4$, 10 KH_2PO_4, 20 taurine, 5 creatine, 0.5 EGTA, and 1 mg/mL bovine serum albumin (BSA) (pH 7.4) three times, then SANCs were dissociated with a transfer pipette by mechanically stirring and pipetting the tissue chunks. Dissociated SANCs were stored at room temperature for study.

Proximity Ligation Assay (PLA)

A Duolink In Situ PLA kit (Sigma-Aldrich) (18) was used to detect RyR2 and cytochrome C oxidase IV (COX IV) complexes, RyR2 and Mfn2, RyR2, and dynamin-related protein 1 (DRP-1) in isolated SANCs. Briefly, cells were fixed with 4% paraformaldehyde and permeabilized with 0.25% Triton X-100. Cells were blocked with 1% BSA for 30 minutes at room temperature and then incubated overnight at 4° C with a specific combination of two primary antibodies in 1% BSA and 0.25% Triton X-100 PBS solution: mouse anti-RyR2 (1:200, Abcam, Cambridge, MA), rabbit anti-mitofusin-2 (1:100, Cell Signaling Technology (CST), Danvers, MA), goat anti-COX-IV (1:200, Santa Cruz Biotechnology, Dallas, TX), rabbit anti-DRP-1 (1:200, CST). Cells were incubated with only one primary antibody as negative controls. PLA probes (anti-mouse MINUS and anti-rabbit PLUS) were used as secondary antibodies to bind to primary antibodies. Ligase was added to cells to allow hybridization with the probes, and polymerase was added for a rolling circle amplification reaction. Coverslips were mounted on a microscope slide with Duolink mounting medium. The fluorescence signal was detected using a Zeiss confocal LSM 700

microscope (Carl Zeiss, Oberkochen, Germany). Images were collected at different optical planes (z-axis step = 0.5 μm). The stack of images for each sample was combined into a single-intensity projection image that was subsequently used to analyze the number of puncta/μm^2 per cell.

Immunofluorescence Confocal Microscopy and Stimulated Emission Depletion (STED) Microscopy

Immunofluorescence labeling was performed as previously described (3). The following primary antibodies were used: mouse anti-RyR2 (1:200, Abcam), rabbit anti-mitofusin-2 (1:100, CST), goat anti-COX-IV (1:200, Santa Cruz), rabbit anti-DRP-1 (1:200, Sigma). The cells were treated with primary antibodies overnight at 4° C. Anti-mouse, or anti-rabbit secondary antibodies (Jackson ImmunoResearch, 1:500 dilution) were used to incubate the cells for 1 hour at room temperature. Slides were imaged using a Zeiss confocal LSM 700 microscope. Control experiments performed by incubation with secondary antibody only did not show positive staining under the same experimental conditions. Identical settings were used for all specimens. MitoTracker Deep Red FM (ThermoFisher Scientific) and tetramethylrhodamine methyl ester (TMRM, ThermoFisher Scientific), both mitochondrial targeted dyes, were used to further determine mitochondrial localization in SANCs. STED microscopy was performed on a Leica STED (TCS SP8 STED 3X) microscope (Leica Microsystems Inc, Buffalo Grove, IL). Deconvolution was performed using Huygens Professional software (Scientific Volume Imaging, Hilversum, Netherlands. Colocalization was analyzed by the Imaris software (Oxford Instruments, Abingdon, UK).

Transmission Electron Microscopy

Once hearts were harvested, the SAN tissues were isolated and placed in a fixative containing 2% paraformaldehyde and 2.5% glutaraldehyde in 0.1M sodium phosphate buffer

overnight. The tissues were then rinsed in 0.1M sodium phosphate buffer twice at 15 minutes each, followed by 45 minutes in 1% osmium tetroxide. After incubation, the tissues were rinsed with ddH_2O two times at 15 minutes each. They then underwent a series of ethanol-induced dehydration, followed by propylene oxide. Pre-infiltration was performed overnight in half propylene oxide, half Poly/Bed Luft resin (Poly/Bed 812, dodecyl succinic anhydride, nadic methyl anhydride, DMP-30). Infiltration was done for 5 hours in 100% Poly/Bed Luft resin. We then embedded the tissues in fresh Poly/Bed Luft resin and allowed the resin to polymerize for 24 hours at 60 °C. Sections of the tissues were taken at approximately 100-nm and collected on copper grids. Sections were stained with 4% aqueous uranyl acetate, rinsed in water, followed by 0.3% lead citrate in 0.1N sodium hydroxide before a final rinse in water. FEI Talos L120C was used to image the sections. Data analysis was done using ImageJ as described (19).

Electron Microscope Tomography

SAN tissue from 3 sham and 3 TAC mice were fixed with 2.5% glutaraldehyde and 2% paraformaldehyde in 0.1 M sodium cacodylate at 37 °C. The tissues were rinsed on ice with 0.1 M sodium cacodylate buffer containing 3 μM $CaCl_2$ and incubated in a mixture of 1% OsO_4, 0.8% potassium ferrocyanide ($C_6FeK_4N_6$), 3 μM $CaCl_2$ in 0.1 M sodium cacodylate for 1 hour on ice. Then, the tissues were washed with ice-cold double-distilled water and stained with 2% uranyl acetate for 1 hour on ice before incubating in increasing ethanol solutions (20%, 50%, 70%, 90% on ice, 3 x 100% at room temperature). Subsequently, the tissues were infiltrated with a mixture of 50% ethanol and 50% Durcupan ACM resin (Fluka) for 6 hours with agitation, and then incubated 3 x 6-12 hours in 100% Durcupan with agitation and polymerized for 48 h at 60 C in an oven.

Semi-thick sections of thickness about 400 nm were cut from the tissue blocks with a Leica ultramicrotome and placed on 200-mesh uncoated thin-bar copper grids. 20-nm colloidal gold particles were deposited on each side of the grid to serve as fiducial cues. The specimens were irradiated for about 15 min before initiating a double-tilt series to limit anisotropic specimen thinning during image collection. During data collection, the illumination was held to near parallel beam conditions. Tilt series were captured using SerialEM (University of Colorado, Boulder, CO) software on a Tecnai HiBase Titan (FEI; Hillsboro, OR) electron microscope operated at 300 kV and 0.81 nm/pixel. Images were recorded with a Gatan 4Kx4K CCD camera. Each double-tilt series consisted of first collecting 121 images taken at 1 degree increment over a range of -60 to +60 degrees followed by rotating the grid 90 degrees and collecting another 121 images with the same tilt increment. To improve the signal-to-noise ratio, 2x binning was performed by averaging a 2x2 x-y pixel box into 1 pixel.

The IMOD package (https://en.wikipedia.org/wiki/IMOD_(software)) was used for alignment, reconstruction, and volume segmentation. R-weighted back projection was used to generate the reconstructions. 53 mitochondrial volumes were generated for sham and 38 mitochondrial volumes for TAC. Volume segmentation of mitochondrial membranes was performed using IMOD by tracing in each of the 1.62 nm-thick *x-y* planes that the object appeared that then created stacks of contours with the Drawing Tools plug-in in IMOD. The traced contours were then surface-rendered by turning contours into meshes to generate a 3D model. The surface-rendered volumes were visualized using 3DMOD.

Measurements of mitochondrial outer, inner boundary, and cristae membrane surface areas and volumes were made within segmented volumes using IMODinfo. Mitochondrial volume density (defined as the volume occupied by mitochondria divided by the cytoplasmic volume),

number of mitochondria per area, crista density (defined as the total membrane surface area of the cristae divided by the volume of the mitochondrion), and mitochondrion-SR distance were measured using ImageJ.

Mitochondrial Ca^{2+} Uptake

SANCs were loaded with X-Rhod-1 AM for 40 minutes at 37°C. They were then plated on coverslips coated with laminin and poly-L-lysine. The cells were initially perfused with 50 µg/mL of Saponin in intracellular solution containing (in mM): 135 KCl, 0 NaCl, 20 HEPES, 5 pyruvate, 2 glutamate, 2 malate, 0.5 KH_2PO_4, 0.5 $MgCl_2$, 15 2,3-butanedione monoxime, 5 EGTA, and 1.86 $CaCl_2$ to yield a free $[Ca^{2+}]_i$ of 100 nM with pH 7.2 (20-22). The solution was then switched to without Saponin. The change in fluorescence intensity monitored mitochondrial Ca^{2+} uptake, normalized to the baseline fluorescence intensity, after adding 1.35 μM Ca^{2+}, 5 μM Ca^{2+}, and 10 μM Ca^{2+}. All measurements were taken at 37°C.

Reactive Oxygen Species (ROS) Production

Intact SANCs were loaded with 0.5 μM MitoSox Red (λ_{ex} = 488 nm, λ_{em} = 510 nm) for 30 minutes at 37°C. ROS generation was monitored under basal conditions and after applying 1 μM isoproterenol in intracellular solution at 37°C as described previously (21, 22)

Mitochondrial ATP Generation

Changes in ATP were monitored using Mg-fluo-4 (Invitrogen, ThermoFisher Scientific) as previously described (3, 23). The positive charge on magnesium binds to the negative phosphates on ATP to stabilize the molecule. Therefore, ATP synthesis causes a decrease in free Mg^{2+}. In contrast, ATP hydrolysis causes more Mg^{2+} to become unbound, leading to an increase in $[Mg^{2+}]_i$ and Mg-fluo-4 signal intensity. SANCs were loaded with 10 μM Mg-fluo-4 (λ_{ex} = 488 nm, λ_{em} = 565-605 nm) for 30 minutes at 37°C. Since we are indirectly monitoring ATP generation,

we first ensure that the changes in Mg^{2+} is due to the changes in ATP generation. We therefore perfused cells with 5 mM pyruvate and 5-mM malate to stimulate complex I-mediated respiration. To stimulate complex II-mediated respiration, 5-mM succinate was supplied. All measurements were taken at 37°C.

Electrophysiology

SANCs were isolated from sham, TAC, and control mice. Spontaneous single-cell APs were recorded in current-clamp mode using the perforated patch configuration at 36 ± 0.5 °C using conventional whole-cell patch-clamp techniques (24). Amphotericin B (240 μg/ml) was added into the pipette solution to form the perforated patch to record APs, using Axopatch 200A amplifier and Digidata 1440 digitizer (Molecular Devices, LLC., Sunnyvale, CA). The signals were filtered at 2 kHz using a 4-pole Bessel filter and digitized at a sampling frequency of 5 kHz. All experiments were performed using 3 M KCl agar bridges, connecting the ground electrode to the recording chamber. Borosilicate glass electrodes were pulled with a P-97 micropipette puller (Sutter Instruments, Novato, CA). The resistance of the electrodes was ~2-3 MΩ when filled with the pipette solutions. Spontaneous APs were recorded in Tyrode's solution with the pipette filled with (in mM): 30 potassium aspartate, 10 NaCl, 10 HEPES, 0.04 $CaCl_2$, 2.0 Mg-ATP, 7.0 phosphocreatine, 0.1 Na-GTP, with pH adjusted to 7.2 with KOH. Carbonyl cyanide p-trifluoro methoxyphenylhydrazone (FCCP, a mitochondrial uncoupler, 1 μM) was applied to sham SANCs after stable recording. To test the effects of ATP-sensitive K^+ channel ($I_{K,ATP}$) inhibition, 100 mM glibenclamide stock solution was made in dimethyl sulfoxide, and then diluted to 10 μM in Tyrode's solution for application.

Data acquisition and analysis were performed using pClamp 10 software (Molecular Devices) and Origin software (OriginLab, Northampton, MA).

Western Blot Analyses

SAN, atrial, and ventricular tissue from the same heart were flash-frozen in liquid nitrogen for western blotting experiments. To compare relative protein expression levels in SAN, atrial, and ventricular tissue, samples of all three tissue types were included in each blot. The same amount of total protein (10 μg) was loaded in each lane. Membranes were blocked in 5% non-fat dry milk (Bio-Rad Laboratories, Hercules, CA) in Tris-buffered saline (TBS) for 1 hour (room temperature) and then incubated with primary antibodies 1:1000 COX IV (Santa Cruz), 1:1000 Mitofusin-2 (CST), 1:1000 DRP-1 (Sigma), and 1:10000 GAPDH (Abcam), all in 5% non-fat dry milk in TBS-T overnight at 4°C.

SAN Whole Mount Fluorescent Immunohistochemistry

The SAN whole-mount fluorescent immunohistochemistry was performed as previously described(25). Hearts from sham and TAC mice were removed and placed into a dissecting dish containing cold 0.01 M PBS. The left and right atrial walls, appendages, and interatrial septum were separated and pinned on a silicone pad in a dissecting dish for fixation for 25 minutes at 4 °C in 4% paraformaldehyde (PFA) solution in 0.01 M PBS (pH 7.4). To decrease tissue autofluorescence, the flattened tissues were cleared using ethanol with dimethylsulfoxide (20%, DMSO), hydrogen peroxide in ethanol (6%, H_2O_2), and dehydrated through a graded ethanol series. The whole-mount preparations were then rehydrated with 10-minute successive washes through a graded ethanol series and permeabilized in 3 × 10-minute changes of 0.01 M PBS containing 0.5% Triton X-100. Nonspecific binding was blocked in PBS containing 5% normal donkey serum and 0.5% Triton X-100. The SAN tissues were incubated in a mixture of anti-HCN4 (rabbit polyclonal, Sigma) and anti-COX IV (goat polyclonal, Santa Cruz) primary antibodies for 48 h at 4 °C. Then they were incubated with Alexa Fluor 488 anti-rabbit and Alexa Fluor 555 anti-

goat secondary antibodies for 4 h in the dark at room temperature. Finally, the SAN tissues were mounted, and STED imaging was performed using the Olympus microscope system (Tokyo, Japan). The whole-mount preparations were imaged using a 10x objective and stitched with Olympus software. Z-stack images were taken and 3D rendering was performed using Imaris software. Zoomed-in images of the SAN head, body, and tail were taken under 40x.

Culture of SANCs

SANCs were isolated from wild type C57BL/6J (WT), *mitofusin-2* knockdown (*Mfn2*-KD), and heart failure (HF) mice as described above. Glass coverslips (25 mm, size #0, Karl Hecht, Sondheim, Germany) were coated with 100x diluted laminin (Life Technologies, Grand Island, NY) and incubated for 4 hours at 37 °C in 5% CO_2. After 4 hours, coverslips were placed in individual wells in a 24-well plate (Falcon, Tewksbury, MA) and washed 3x with sterile PBS (in mM: 137 NaCl, 2.7 KCl, 10 Na_2HPO_4, 1.8 KH_2PO_4, pH = 7.4). Isolated SANCs were resuspended in M1018 medium (10.7 g/L) supplemented with 1x penicillin-streptomycin-glutamate (PSG), 4 mM $NaHCO_3$, 10 mM HEPES, 10% fetal bovine serum (FBS), 20 μM (S) nitro-blebbistatin and plated on the laminin pre-coated coverslips and incubated for 4 hours at 37 °C in 5% CO_2, before the media was replaced with serum-free M1018 (16).

Adenoviral Transfection of Protein Kinase A (PKA) Biosensors in SANCs and Confocal Imaging

For infection of adult mouse SANCs, the serum-free M1018 media was replaced with 500 μL of the serum-containing medium with recombinant adenoviruses encoding for targeted FRET-based biosensors. Accordingly, we employed the SR-targeted AKAR (SR-AKAR3). Cells infected with the desired adenoviral vectors were incubated at 37° C with 5% CO_2 for 36 to 40 hours.

After 40 hr infection, SANCs were fixed with 4% PFA for Immunofluorescence labeling. Cells were then washed with PBS (3 x 10 minutes). Cells were permeabilized for 20 minutes with 0.1% Triton X-100% and then blocked with 5% donkey serum for 1 hour at room temperature. The following primary antibodies were used to incubate cells overnight at 4°C.

All the antibodies used were diluted in a 5% donkey serum blocking solution. Cells were then washed with PBS (3 x 10 minutes). Coverslips were mounted on the slides with ProLong Diamond Antifade Mountant (Thermo Fisher Scientific Inc., Waltham, MA). Identical settings and acquisition parameters were used for all specimens. Cells were sequentially imaged using a Zeiss 900 confocal laser-scanning microscope equipped with an Airyscan detector module, a Plan-Apo 63 × 1.4 NA oil-immersion objective, and 488/561 lasers. Images were background subtracted, pseudo-colored, and analyzed offline using ImageJ.

Whole-cell Ca^{2+} Transient Measurements

IonOptix contraction system (IonOptix LLC, Westwood, MA) was used to detect spontaneous Ca^{2+} transients (CaT) from single isolated SANCs as we have previously described (11, 12, 26). Freshly isolated SAN cells were loaded with 5 μM Fluo-4 AM (F14201, ThermoFisher Scientific) for 15 minutes at RT. Cells were then perfused with Tyrode's solution (36 ± 0.5 °C) continuously. Fluo-4 loaded SANCs were excited by a selected wavelength of 500 ± 10 nm, and the emitted fluorescent signal was filtered using a band-pass filter of 535 ± 15 nm and collected by a photomultiplier. The maximal Fluo-4 fluorescence (F) was measured at peak amplitude and was normalized to the average of baseline fluorescence (F_0). Background fluorescence was subtracted for each recording. To test the effects of mitochondria-targeted antioxidant on CaT, 10 mM MitoTEMPO stock solution was made in deionized water, and diluted to 1 μM in Tyrode’s solution for the treatment of the isolated cells (30 min at room temperature).

Local Ca^{2+} Release (LCR) using Confocal Line Scanning

LCR was quantified as previously described (27). Freshly isolated SAN cells were loaded with 5 μM Fluo-4 AM for 15 minutes at room temperature. Cells were then perfused with Tyrode's solution (36 ± 0.5 °C). Line-scan images across the whole SANCs were obtained to quantify local Ca^{2+} signals with 488 nm excitation and 510 nm emission. Pixel time was 0.76 μs; line time was 0.91 bms. Pinhole was set at 1.00 AU (Airy unit).

Fluorescence Resonance Energy Transfer (FRET) Imaging and Quantification

After a 40 hour infection, the media was changed to serum-free media. On imaging day, glass coverslips were transferred to a glass-bottom culture dish (MatTek, Ashland, MA) containing 3 mL PBS at room temperature. A Leica DMI3000B inverted fluorescence microscope (Leica Biosystems, Buffalo Grove, IL) equipped with a Hamamatsu Orca-Flash 4.0 digital camera (Bridgewater, NJ) controlled by Metaflor software (Molecular Devices) acquired phase contrast, CFP480, and FRET images. Phase contrast and CFP480 images were collected with 20x and 40x oil immersion objective lenses, while FRET images were gathered using only the 40x oil immersion objective lens. Images for FRET analysis were recorded by exciting the donor fluorophore at 430-455nm and measuring emission fluorescence with two filters (475DF40 for cyan and 535DF25 for yellow). Images were subjected to background subtraction and acquired every 30 seconds with an exposure time of 200 ms for each channel. The donor/acceptor FRET ratio was calculated and normalized to the ratio value of baseline with isoproterenol. CFP480 images were acquired by exciting the donor fluorophore at 430-455 nm and measuring emission fluorescence with the 475DF40 filter for 25 ms. The FRET ratio changes graphed the average response curves and normalized isoproterenol responoses to forskolin+IBMX. The binding of

cAMP to each FRET biosensor increased the YFP/CFP and was interpreted to increase cAMP levels. Experiments were performed at room temperature (22-25 °C).

Statistical Analysis

All data are reported as mean ± standard error unless otherwise stated. Statistical significance was determined using the student paired t-test. A value of $p<0.05$ was considered statistically significant.

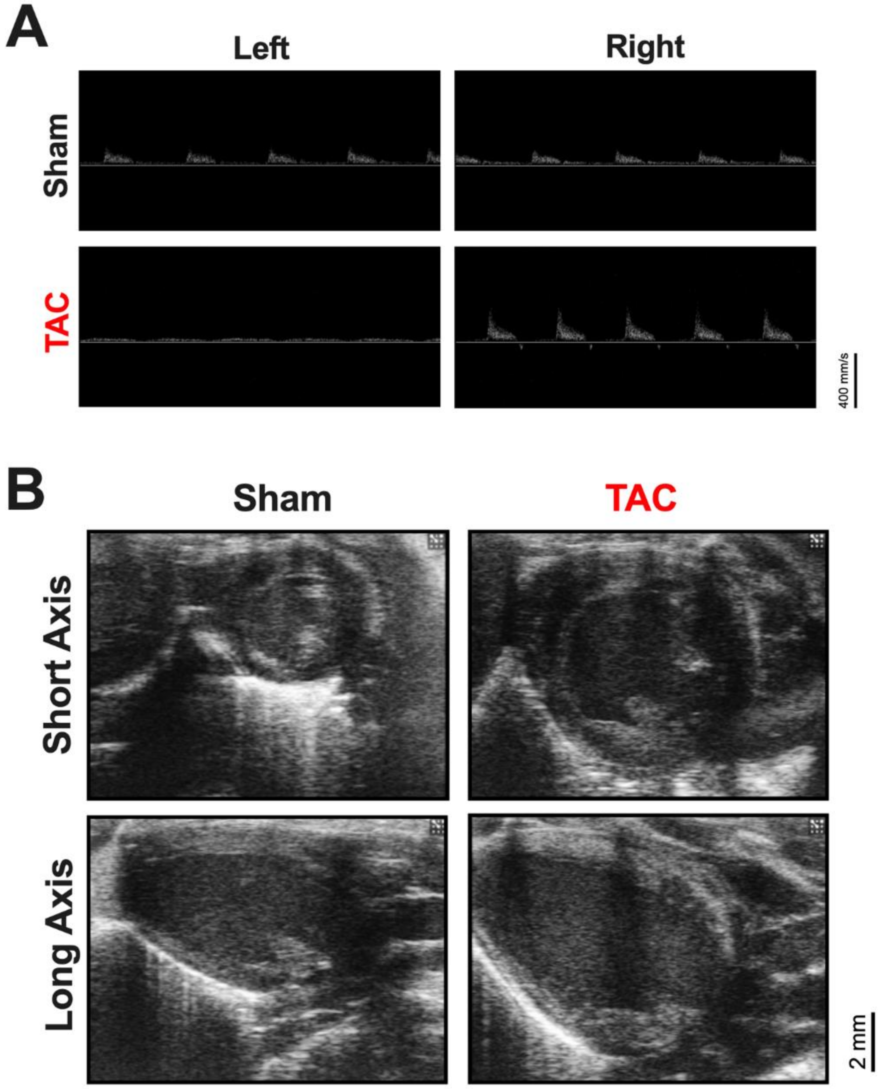

Fig. S1. Echocardiography validation. A) Representative images of blood flow velocity taken at the left and right carotid arteries of sham and TAC mice, showing evidence of aortic constriction. **B**) Representative images of parasternal short axis and long-axis images of sham and TAC mice, demonstrating enlargement of the hearts in TAC mice.

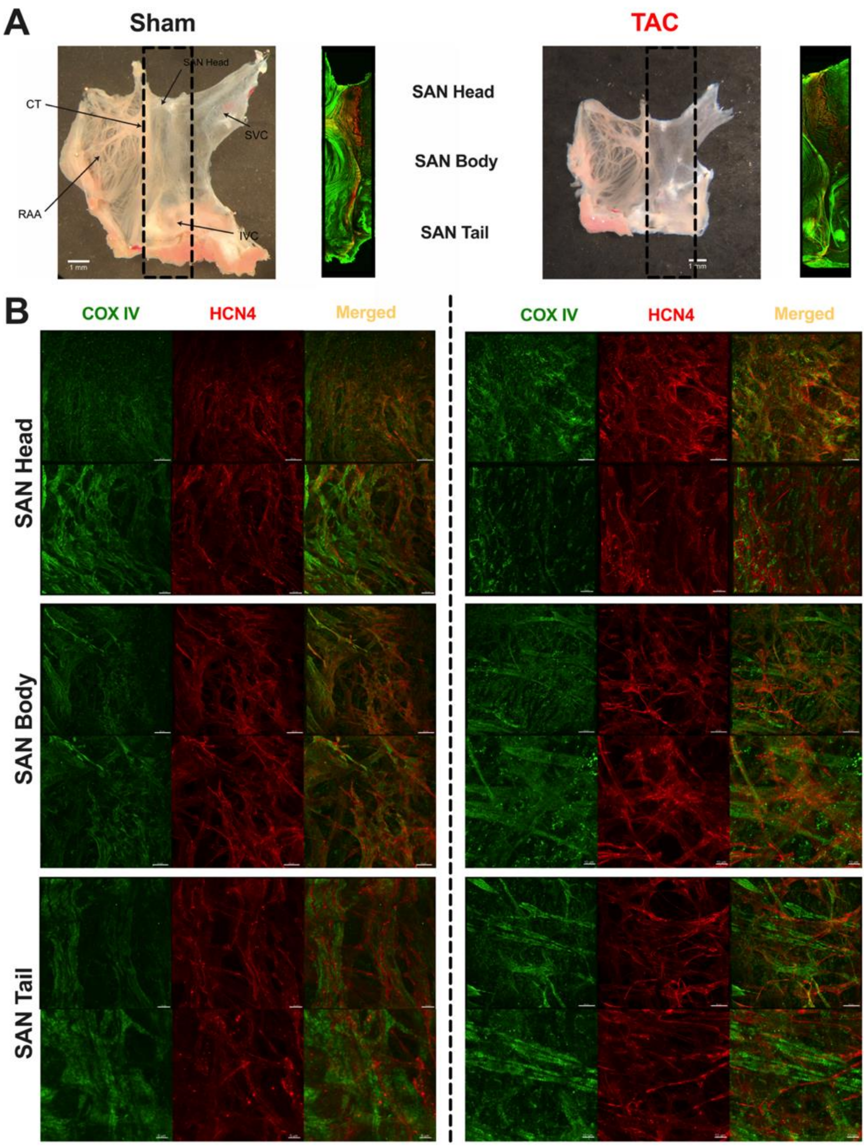

Fig. S2. High density of mitochondria within the SAN tissue. Whole-mounted SAN tissue from both sham and TAC mice examined with STED imaging. Panel **A**) shows tissue preparation and the whole SAN sections labeled with COX IV (green) and HCN4 (red). **B**) Images of the SAN head, body, and tail for the two groups. Zoomed-in and zoomed-out images are shown for each region. Scale bars are 5 μm.

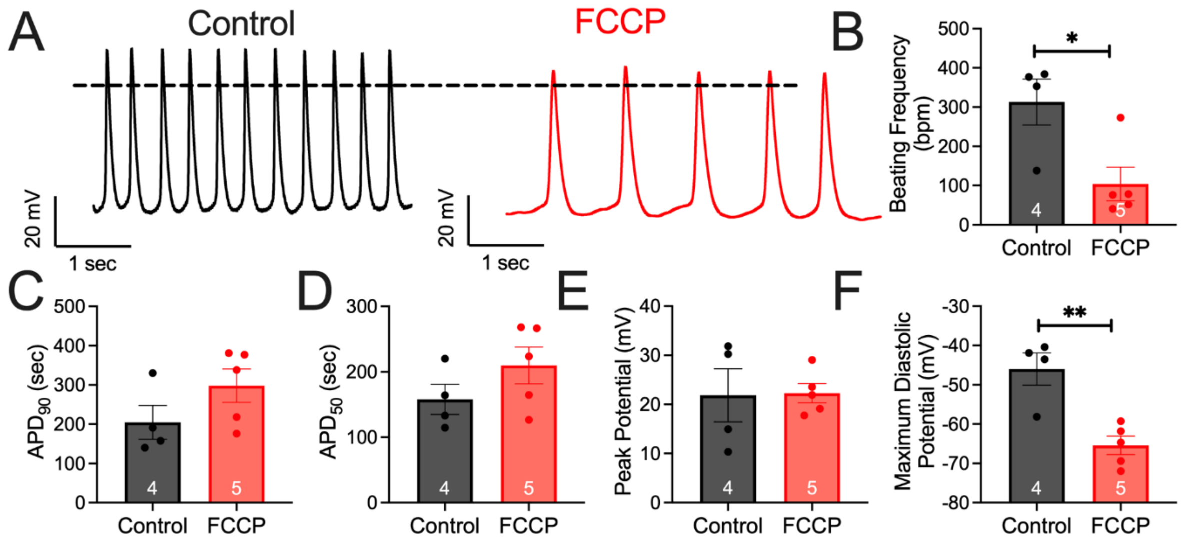

Fig. S3. FCCP reduces SAN automaticity in SANCs. A) Representative tracings show the effects of 1 μM carbonyl cyanide-p-trifluoromethoxyphenylhydrazone (FCCP) on SAN cells. Summary data of **B**) beating frequency, **C**) action potential duration at 90% repolarization (APD90), **D**) APD50, **E**) peak potential, and **F**) maximum diastolic potential are shown. Data are expressed as mean ± SEM. n=3 mice for all; n=4 SANCs for control, and n=5 SANCs for FCCP. **p*<0.05, ***p*<0.01.

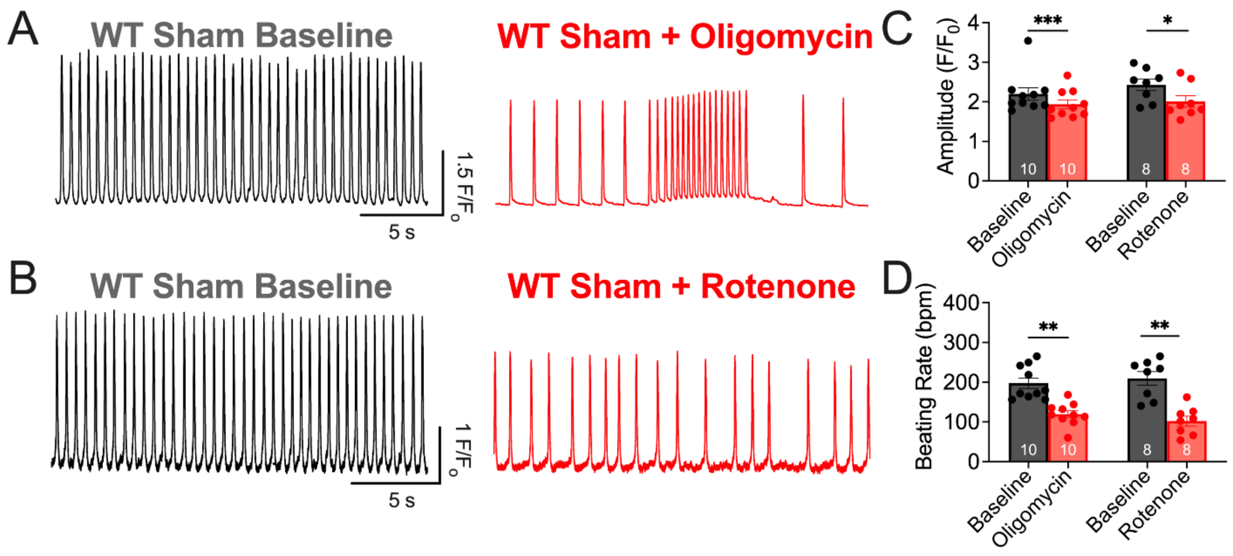

Fig. S4. WT SANCs exhibited impaired CaT in the presence of mitochondrial toxins. A) Representative traces of WT SANC at baseline and after applying oligomycin are shown. **B**) Representative traces of WT SANC at baseline and after the application of rotenone are displayed. Summary data show the **C**) amplitude and **D**) beating rate of the CaT. n=3-4 mice; n=individual number of cells are displayed in the graphs. Data are expressed as mean ± SEM. **p*<0.05, ***p*<0.01, ****p*<0.001.

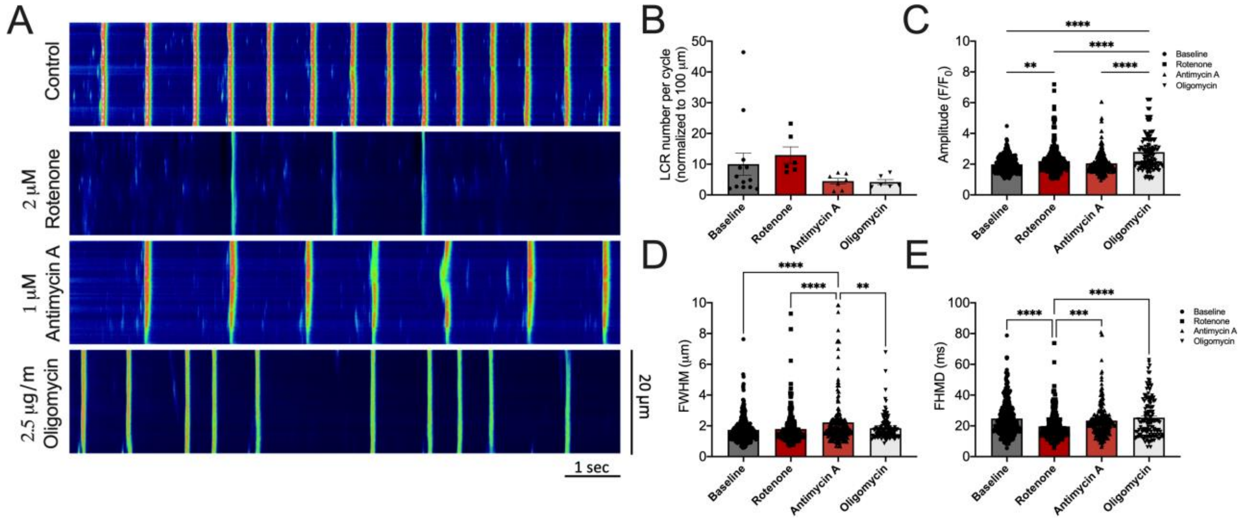

Fig. S5. SANCs exhibited impaired local Ca^{2+} release in the presence of mitochondrial toxins. A) Representative images of isolated SANCs perfused with no toxin, rotenone, antimycin A, or oligomycin. **B**) Summary data of LCR number per cycle, **C**) amplitude, full width at half maximum (FWHM), and **E**) full duration at half maximum (FDHM). Data are expressed as mean ± SEM. $^{**}p<0.01$, $^{***}p<0.001$, $^{****}p<0.0001$.

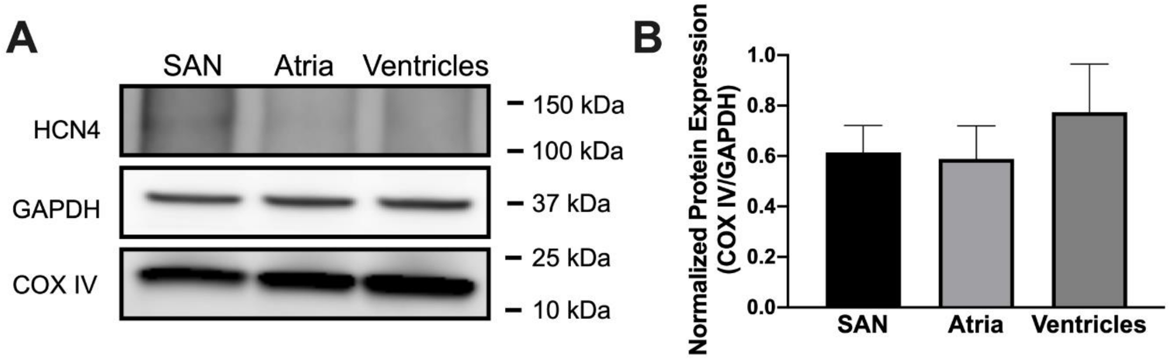

Fig. S6. Cytochrome c oxidase subunit IV (COX IV) is highly expressed in SAN, atrial, and ventricular tissues. A) Representative western blot images show similar levels of COX IV, a mitochondrial protein, in SAN, atrial and ventricular tissues. **B**) Similar levels were seen when COX IV was normalized to GAPDH. n=3 mice for all groups.

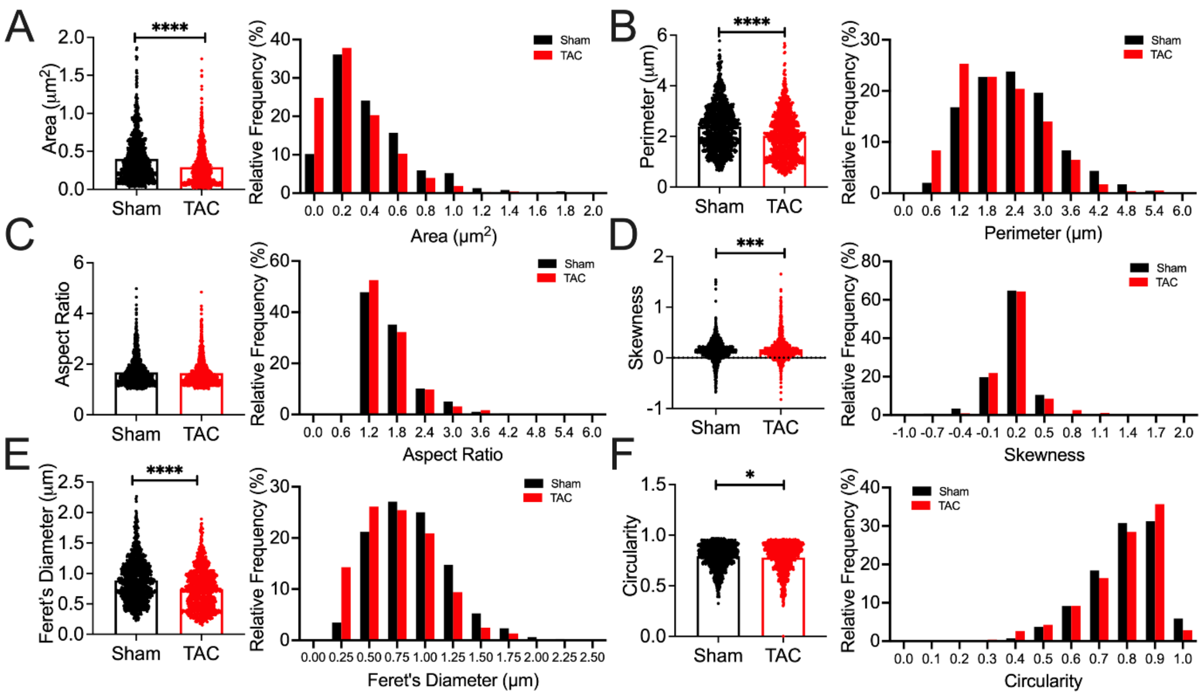

Fig. S7. Mitochondrial morphology and distribution. To assess morphological parameters, we examined 100 nm sections of SAN tissues under the TEM. Summary data and histogram distribution of **A**) area, **B**) perimeter, **C**) aspect ratio, **D**) skewness, **E**) Feret's diameter, and **F**) circularity are shown. EM tomography reviewed distinct morphological changes in mitochondria. n=1100 mitochondria for the sham group and n=980 mitochondria for the TAC group**.** Quantification of **G**) area, **H**) perimeter, **I)** aspect ratio, **J)** skewness, **K)** Feret's diameter, **L)** and circularity is displayed. *p<0.05, ***p<0.001, and ****p<0.0001.

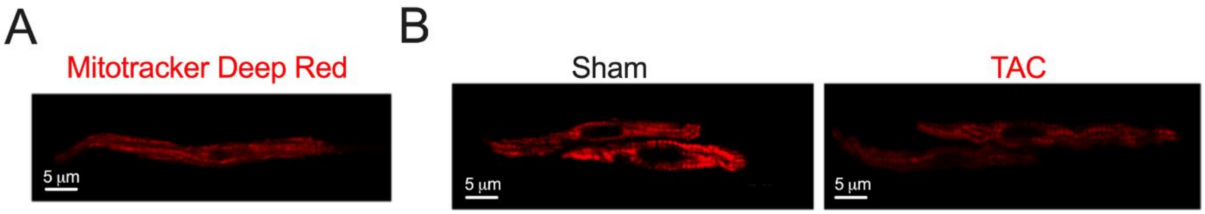

Fig. S8. Distribution of mitochondria. **A**) A representative image of a SANC stained with MitoTracker Deep Red FM dye. **B**) Images showing SANCs in sham and TAC group loaded with 5 nM tetramethylrhodamine, methyl ester (TMRM).

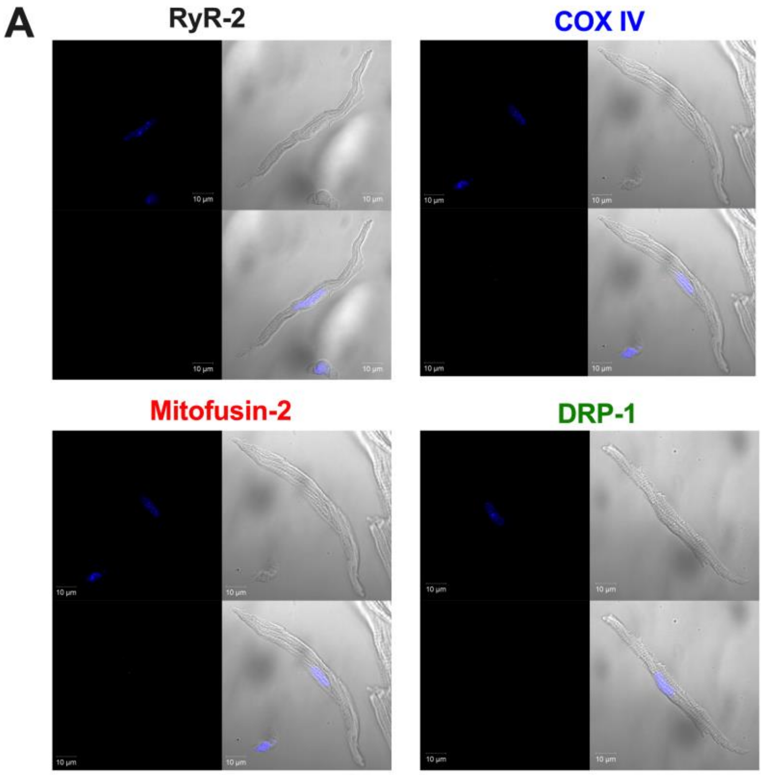

Fig. S9. A) Negative control images for PLA experiments using one antibody (RyR2, COX IV, Mitofusin-2, or DRP-1).

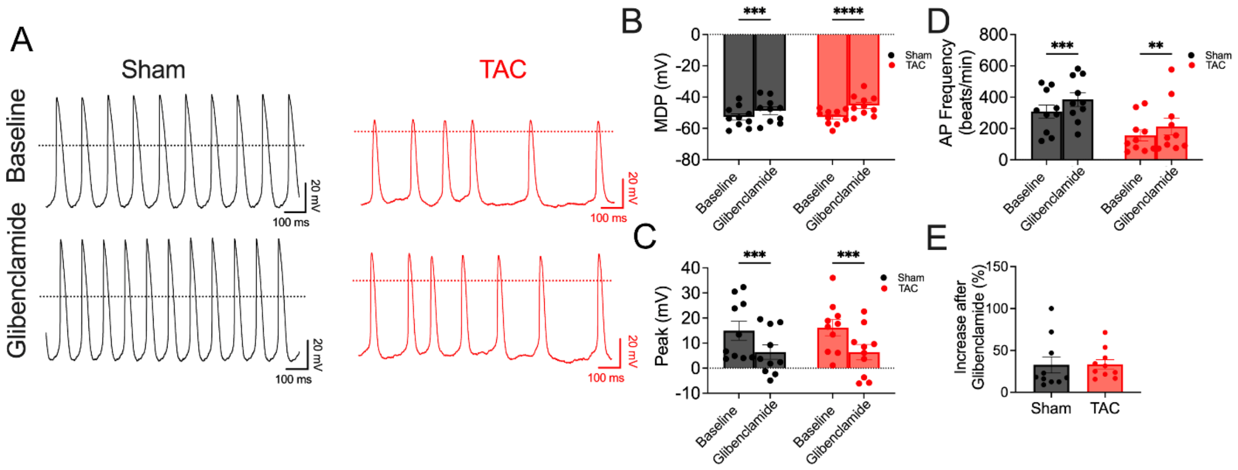

Fig. S10. Glibenclamide increases AP frequency in SANCs. **A**) Representative AP recordings from sham compared to HF SANCs at baseline and after 10 μM glibenclamide. Summary data for **B**) Maximum diastolic potentials (MDP in mV),) Peak potentials (in mV), **D**) AP frequency at baseline and after glibenclamide for sham compared to HF SANCs. E) Percent increase in AP frequency after glibencamide. Data are expressed as mean ± SEM. Two-way ANOVA with appropriate post-hoc test was performed. **p<0.01 and ***p<0.001, n = 10 cells for each group.

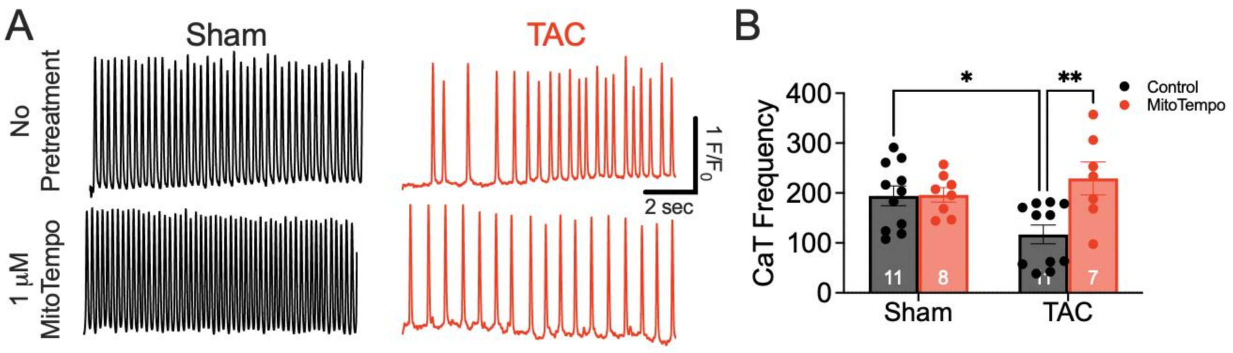

Fig. S11. **Pretreatment with MitoTempo normalized CaT frequency in HF SANCs**. **A**) Representative recordings of CaTs from sham and TAC SANCs, with and without 30-minute pretreatment with 1 μM MitoTempo. **B**) Summary data of CaT frequency. Data are expressed as mean ± SEM. Two-way ANOVA analysis with appropriate post-hoc test was performed. **p*<0.05, **p<0.01, n = 7-11 cells.

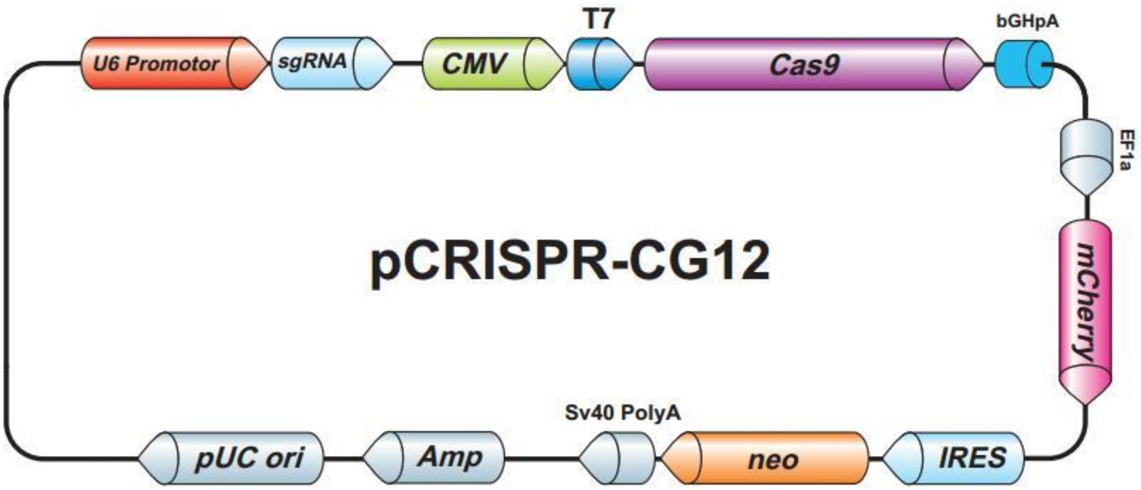

Fig. S12. Single-guide RNAs (sgRNAs) expression clones targeting *Mfn2* gene (pNV-sgRNA-Cas9-2A-GFP vector from GeneCopoeia, Inc) and liposomes as carriers for gene delivery will be used. The vector contains Cas9 endonuclease and mCherry reporter genes. Scrambled sgRNAs will be used for control. The vector-loaded liposomes are delivered to the epicardial surface of the SAN.

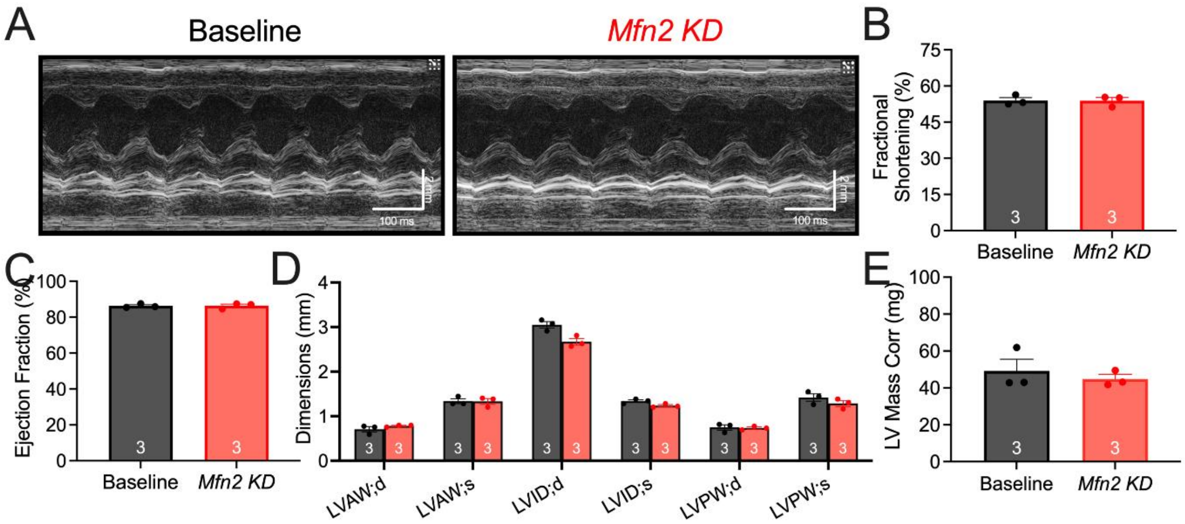

Fig. S13. No evidence of cardiac dysfunction in SAN-specific *Mfn KD* mice. Even though *Mfn2 KD* mice experienced SAN dysfunction, there was no evidence of cardiac dysfunction. **A**) Representative M-mode images at the parasternal short axis are depicted from baseline and after *Mfn2 KD*. Summary data for **B)** fractional shortening, **C)** ejection fraction, D) left ventricular dimensions in mm (LVAW;d and LVAW;s, left ventricular anterior wall in diastole and systole, respectively, LVID;d and LVID;s, left ventricular internal dimension in diastole and systole, respectively, LVPW;d and LV) fractional shortening are shown. **E**) Corrected LV mass. *p= *NS*. The numbers shown within the bar graphs represent the numbers of animals.

	Sham (n=14)	TAC (n=22)	p value
Heart Rate (bpm)	596±9	552±14	<0.05
LV Mass Corr (mg)	70.27±4.03	176.44±3.71	<0.0001
LVAW;d (mm)	0.822±0.024	1.137±0.031	<0.0001
LVAW;s (mm)	1.448±0.032	1.427±0.049	<0.0001
LVID;d (mm)	3.313±0.080	4.565±0.092	<0.0001
LVID;s (mm)	1.532±0.053	3.774±0.122	<0.0001
LVPW;d (mm)	0.800±0.024	1.133±0.026	<0.0001
LVPW;s (mm)	1.447±0.023	1.366±0.048	<0.0001
CO (ml/min)	24.14±1.22	20.40±0.94	<0.05
Ejection Fraction (%)	85.51±0.08	36.57±2.34	<0.0001
Fractional Shortening (%)	54.21±0.89	17.74±1.29	<0.0001
LV Vol;d (µl)	45.09±2.62	96.803±4.41	<0.0001
LV Vol;s (µl)	6.60±0.57	62.99±4.40	<0.0001

Table S1. Echocardiography parameters. LV Mass Corr = left ventricular mass corrected. LVAW;d and LVAW;s = left anterior ventricular wall at diastole and at systole. LVID;d and LVID;s = left ventricular inner diameter at diastole and systole. LVPW;d and LVPW;s = left posterior ventricular wall at diastole and systole. CO = cardiac output. LV Vol;d and LV Vol;s = left ventricular volume at diastole and systole.

	Sham (n=1100)	TAC (n=980)	p value
Area (μm^2)	0.401±0.009	0.291±0.008	<0.0001
Perimeter (µm)	2.39±0.03	2.03±0.03	<0.0001
Circularity	0.792±0.004	0.780±0.004	<0.05
Aspect ratio	1.67±0.02	1.64±0.02	NS
Skewness	0.140±0.006	0.166±0.007	<0.001
Roundness	0.650±0.005	0.659±0.005	NS
Feret's Diameter (µm)	0.887±0.011	0.744±0.011	<0.0001

Table S2. Mitochondrial characteristics.

SI References:

1. Helassa N, Podor B, Fine A, & Török K (2016) Design and mechanistic insight into ultrafast calcium indicators for monitoring intracellular calcium dynamics. *Sci Rep* 6(1):38276.
2. Lee FK, *et al.* (2021) Genetically engineered mice for combinatorial cardiovascular optobiology. *Elife* 10.
3. Thai PN, *et al.* (2018) Cardiac-specific Conditional Knockout of the 18-kDa Mitochondrial Translocator Protein Protects from Pressure Overload Induced Heart Failure. *Sci Rep* 8(1):16213-16213.
4. Sirish P, *et al.* (2013) Unique mechanistic insights into the beneficial effects of soluble epoxide hydrolase inhibitors in the prevention of cardiac fibrosis. *Proc Natl Acad Sci U S A* 110(14):5618-5623.
5. Doudna JA & Sontheimer EJ (2014) Methods in Enzymology. The use of CRISPR/Cas9, ZFNs, and TALENs in generating site-specific genome alterations. Preface. *Methods Enzymol* 546:xix-xx.
6. Gilbert LA, *et al.* (2013) CRISPR-mediated modular RNA-guided regulation of transcription in eukaryotes. *Cell* 154(2):442-451.
7. Lo TW, *et al.* (2013) Precise and heritable genome editing in evolutionarily diverse nematodes using TALENs and CRISPR/Cas9 to engineer insertions and deletions. *Genetics* 195(2):331-348.
8. Kikuchi K, McDonald AD, Sasano T, & Donahue JK (2005) Targeted modification of atrial electrophysiology by homogeneous transmural atrial gene transfer. *Circulation* 111(3):264-270.

9. Swaminathan PD, *et al.* (2011) Oxidized CaMKII causes cardiac sinus node dysfunction in mice. *J Clin Invest* 121(8):3277-3288.

10. Lu L, *et al.* (2015) Regulation of gene transcription by voltage-gated L-type calcium channel, $Ca_v1.3$. *J Biol Chem* 290(8):4663-4676.

11. Sirish P, *et al.* (2017) Action Potential Shortening and Impairment of Cardiac Function by Ablation of Slc26a6. *Circ Arrhythm Electrophysiol* 10(10).

12. Zhang XD, *et al.* (2021) Prestin amplifies cardiac motor functions. *Cell Rep* 35(5):109097.

13. Au - Cesarovic N, Au - Jirkof P, Au - Rettich A, & Au - Arras M (2011) Implantation of Radiotelemetry Transmitters Yielding Data on ECG, Heart Rate, Core Body Temperature and Activity in Free-moving Laboratory Mice. *JoVE* (57):e3260.

14. Zhang X-D, *et al.* (2014) Critical roles of a small conductance Ca^{2+}-activated K^+ channel (SK3) in the repolarization process of atrial myocytes. *Cardiovascular Research* 101(2):317-325.

15. Fenske S, *et al.* (2016) Comprehensive multilevel in vivo and in vitro analysis of heart rate fluctuations in mice by ECG telemetry and electrophysiology. *Nat Protoc* 11(1):61-86.

16. Reddy GR, *et al.* (2022) Deciphering cellular signals in adult mouse sinoatrial node cells. *iScience* 25(1):103693.

17. Zhang Z, *et al.* (2002) Functional Roles of $Ca_v1.3$ (α_{1D}) calcium channel in sinoatrial nodes: insight gained using gene-targeted null mutant mice. *Circ Res* 90(9):981-987.

18. Fredriksson S, *et al.* (2002) Protein detection using proximity-dependent DNA ligation assays. *Nat Biotechnol* 20(5):473-477.

19. Picard M, White K, & Turnbull DM (2013) Mitochondrial morphology, topology, and membrane interactions in skeletal muscle: a quantitative three-dimensional electron microscopy study. *J Appl Physiol (1985)* 114(2):161-171.

20. Dedkova EN, Seidlmayer LK, & Blatter LA (2013) Mitochondria-mediated cardioprotection by trimetazidine in rabbit heart failure. *J Mol Cell Cardiol* 59:41-54.

21. Seidlmayer LK, Gomez-Garcia MR, Blatter LA, Pavlov E, & Dedkova EN (2012) Inorganic polyphosphate is a potent activator of the mitochondrial permeability transition pore in cardiac myocytes. *J Gen Physiol* 139(5):321-331.

22. Dedkova EN & Blatter LA (2012) Measuring mitochondrial function in intact cardiac myocytes. *J Mol Cell Cardiol* 52(1):48-61.

23. Seidlmayer LK, *et al.* (2019) Dual role of inorganic polyphosphate in cardiac myocytes: The importance of polyP chain length for energy metabolism and mPTP activation. *Arch Biochem Biophys* 662:177-189.

24. Hamill OP, Marty A, Neher E, Sakmann B, & Sigworth FJ (1981) Improved patch-clamp techniques for high-resolution current recording from cells and cell-free membrane patches. *Pflugers Arch* 391(2):85-100.

25. Pauza DH, *et al.* (2014) Innervation of sinoatrial nodal cardiomyocytes in mouse. A combined approach using immunofluorescent and electron microscopy. *J Mol Cell Cardiol* 75:188-197.

26. Lyu Y, *et al.* (2022) Beat-to-beat dynamic regulation of intracellular pH in cardiomyocytes. *iScience* 25(1):103624.

27. Tagirova Sirenko S, *et al.* (2021) Self-Similar Synchronization of Calcium and Membrane Potential Transitions During Action Potential Cycles Predict Heart Rate Across Species. *JACC: Clinical Electrophysiology* 7(11):1331-1344.
